# Distinct patterns of plaque and microglia glycosylation in Alzheimer's disease

**DOI:** 10.1111/bpa.13267

**Published:** 2024-05-09

**Authors:** Caitlyn Fastenau, Madison Bunce, Mallory Keating, Jessica Wickline, Sarah C. Hopp, Kevin F. Bieniek

**Affiliations:** ^1^ Department of Pharmacology University of Texas Health Science Center San Antonio San Antonio Texas USA; ^2^ Glenn Biggs Institute for Alzheimer's and Neurodegenerative Diseases University of Texas Health Science Center San Antonio San Antonio Texas USA; ^3^ Department of Pathology and Laboratory Medicine University of Texas Health Science Center San Antonio San Antonio Texas USA

**Keywords:** Alzheimer's disease, digital pathology, glycosylation, microglia, senile plaque, sialylation

## Abstract

Glycosylation is the most common form of post‐translational modification in the brain. Aberrant glycosylation has been observed in cerebrospinal fluid and brain tissue of Alzheimer's disease (AD) cases, including dysregulation of terminal sialic acid (SA) modifications. While alterations in sialylation have been identified in AD, the localization of SA modifications on cellular or aggregate‐associated glycans is largely unknown because of limited spatial resolution of commonly utilized methods. The present study aims to overcome these limitations with novel combinations of histologic techniques to characterize the sialylation landscape of *O*‐ and *N*‐linked glycans in autopsy‐confirmed AD post‐mortem brain tissue. Sialylated glycans facilitate important cellular functions including cell‐to‐cell interaction, cell migration, cell adhesion, immune regulation, and membrane excitability. Previous studies have not investigated both *N*‐ and *O*‐linked sialylated glycans in neurodegeneration. In this study, the location and distribution of sialylated glycans were evaluated in three brain regions (frontal cortex, hippocampus, and cerebellum) from 10 AD cases using quantitative digital pathology techniques. Notably, we found significantly greater *N*‐sialylation of the Aβ plaque microenvironment compared with *O*‐sialylation. Plaque‐associated microglia displayed the most intense *N*‐sialylation proximal to plaque pathology. Further analyses revealed distinct differences in the levels of *N*‐ and *O*‐sialylation between cored and diffuse Aβ plaque morphologies. Interestingly, phosphorylated tau pathology led to a slight increase in *N*‐sialylation and no influence of *O*‐sialylation in these AD brains. Confirming our previous observations in mice with novel histologic approach, these findings support microglia sialylation appears to have a relationship with AD protein aggregates while providing potential targets for therapeutic strategies.

## Graphical abstract

Our study provides an innovative approach to histologically probe challenging *N*‐linked and *O*‐linked post‐translational modifications of sialic acid at the tissue localize modifications proximal to Alzheimer's Disease pathological aggregates.

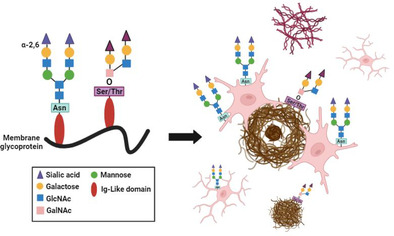

## 1 INTRODUCTION

Alzheimer's disease (AD) is the most common form of dementia and devastating neurodegenerative disease [1]. AD is characterized by two pathognomonic features: extracellular amyloid‐beta (Aβ) plaques aggregates and intraneuronal phosphorylated tau neurofibrillary tangle (NFT) inclusion bodies [2]. Dysregulation of microglia, the innate immune cells of the central nervous system, is also a key feature of AD pathology [3]. Under normal conditions, microglia prune synapses, surveil the brain for threats to homeostasis, and clear debris [4]. During threats to homeostasis, microglia alter their phenotype to react to changes in the microenvironment and to maintain homeostasis [5]. In AD, microglia react to the pathological aggregates in the microenvironment, altering functions including phagocytosis and secretion of cytokines that can positively and negatively influence neuropathology [6]. Interestingly, the terminal sugar residue on glycoproteins and glycolipids, sialic acid (SA), plays a role in regulation of microglia activities including phagocytosis and secretion of cytokines through several different pathways, including activation of SA receptors (Siglecs) like CD22 and CD33 that are associated with aging and AD risk [7, 8, 9] Therefore, investigating localization of sialylated glycans within the AD brain is a crucial first step to understand the relationship between SA and AD neuropathology.

Sialylated glycans can be modified with terminal *N*‐ and *O*‐linked SA residues in a process called sialylation [10, 11]. *N*‐linked sialylation includes various bond types of α‐2,6, α‐2,3, and α‐2,8. Each SA residue participates in different cellular functions. In particular, α‐2,6 SA plays functional roles in cell adhesion, neurodevelopment, and immune regulation [12, 13, 14, 15]. Importantly, changes in sialylation has been previously implicated in AD. Comparing cerebrospinal fluid (CSF) from AD patients and patients with mild cognitive impairment (MCI), AD patients had a significantly reduced degree of sialylation which was predictive of MCI conversion to AD [16], while blood levels of SA decreased in AD patients [17]. More recently, quantitative glycoproteomic studies of *N*‐glycosylation in CSF of AD and healthy cases show SA bond‐specific increases in AD cases [18] and analysis of *O*‐glycosylation in CSF of neurologically normal individuals show an increase in *O*‐glycosylated glycoproteins during early disease progression compared with nonsialylated glycoproteins or other modifications [19]. Taken together, these quantitative studies provide evidence for changes in sialylated glycans in AD compared with neurologically normal controls. Functionally, hyperglycosylated proteins in AD are associated with inflammatory, cell signaling, and receptor binding pathways, while hypoglycosylated proteins related to neuronal function [20]. Specific SA residues are enriched in both the hippocampus and cortex of AD cases compared with controls [21]. *O*‐glycosylation is a regulator of tau phosphorylation in vitro and in vivo, with decreased *O*‐glycosylation in AD brains [22]. Previous work has been subject to the spatial and temporal limitations of mass spectrometry techniques. To localize SA residues at a cellular level, our previous study found increased α‐2,6 *N*‐linked SA localized within the Aβ plaque microenvironment in the 5XFAD mouse model of amyloid pathology. Further, we discovered α‐2,6 but not α‐2,3 *N*‐sialylation is most prominently found on Aβ plaque‐associated microglia and not localized to the Aβ plaque itself in this mouse model [23]. The present study validates this murine observation in human neurodegenerative disease and expands these analyses to better understand both *N*‐ and *O*‐sialylation in postmortem AD cases with both amyloid and tau pathology. To do so, we examined the spatial relationship of *N*‐ and *O*‐linked SA modifications, microglia, and pathological aggregates in the middle frontal gyrus, hippocampus, and cerebellum in human AD cases. Additionally, we validated methods to probe *O*‐linked SA modifications in human brain tissue.

## 2 MATERIALS AND METHODS

### 2.1 Human brain tissue

Ten autopsied brains from the UTHSCSA Glenn Biggs Institute Brain Bank were selected for use in this study. The 10 cases were assigned a Thal phase for Aβ plaque pathology, Braak staging for tau pathology, and the semiquantitative score of neuritic plaques from the Consortium to Establish a Registry for Alzheimer's Disease (CERAD) [24, 25, 26, 27, 28] to ascertain AD neuropathological change (ADNC) scores based on consensus criteria [29]. This cohort included high ADNC (*N* = 7), intermediate (*N* = 1), and low (*N* = 2) composite scores. Consent and demographic information were obtained from donor next‐of‐kin prior to brain autopsy demographics, pathological staging, and ADNC categories are available in Table 1. All patients presented antemortem with indications of AD.

**TABLE 1:** Cohort demographics.

Case #	Age	Sex	ADNC	Braak stage	Thal phase	CERAD
1	61	M	High	6	5	C3
2	62	M	High	6	5	C3
3	62	F	High	6	5	C3
4	64	F	Low	1	0	C0
5	72	M	High	5	4	C3
6	74	M	High	6	5	C3
7	76	M	High	6	5	C3
8	79	M	Int	3	3	C0
9	86	F	Low	3	2	C0
10	87	F	High	6	5	C2

*Note*: Description of the AD case demographics including case number, age, sex, ADNC category, Braak stage, Thal phase, and CERAD score.

Abbreviations: AD, Alzheimer's disease; ADNC, Alzheimer's disease neuropathological change.

### 2.2 Tissue processing and serial sections

**FIGURE 1:**
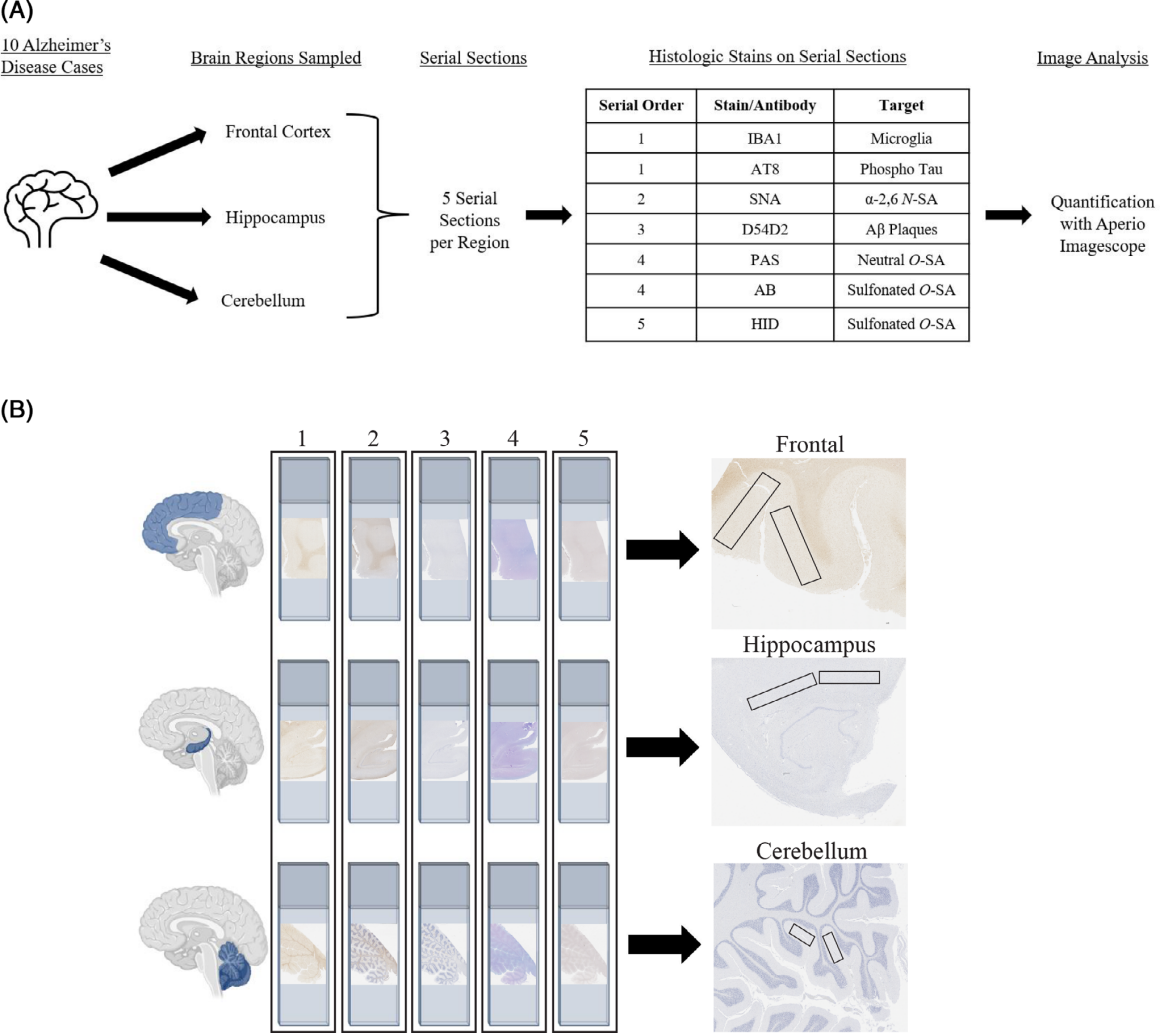
Study design, sampling, and analysis methodology. Schematic overview of the study design. (A) Flowchart of study design, tissue sampling, and histologic probes. (B) Graphical depiction of histologic staining paradigm and region‐specific sampling. The numbers on the slide columns correspond to the histologic probes in (A).

Left hemibrains were fixed in 10% neutral buffered formalin (Leica cat. #3800540) for at least 1 month. Fixed tissue was sectioned coronally, processed for 28 h (Leica ASP300S Tissue Processor), and paraffin‐embedded into tissue blocks using Histoplast Paraffin wax (ThermoFisher HistoStar Embedding Workstation). The regions sampled from the 10 cases included the middle frontal gyrus, hippocampus at the level of the lateral geniculate nucleus, and cerebellum at the level of the dentate nucleus. Following tissue processing, paraffin blocks were cut into five consecutive serial sections at a thickness of 5 μm on a microtome (Leica HistoCore Autocut) resulting in 150 total slides. Tissue sections were mounted on positively charged slides (Fisherbrand Superfrost Plus Microscope Slides) and baked at 60°C overnight for histology. Serial section order and staining paradigm is illustrated in Figure 1.

### 2.3 Immunohistochemistry (IHC) for Aβ plaques

Aβ plaques were visualized with a standard IHC protocol. Slides were deparaffinized with serial washes in xylene (3×, 5 min.), 100% ethanol (2×, 2 min.), 95% ethanol (1×, 2 min.) and flushed in DiH_2_O (Leica ST5010 Autostainer XL). Following deparaffinization, tissue slides were steamed in DiH_2_0 for 30 min and placed in a solution of Tris Buffered Saline with 0.1% Tween 20 detergent. Slides were loaded on LabVision 480S Autostainer where tissue was quenched in 3% H_2_O_2_ for 10 min and blocked in 2.5% normal goat serum (Sigma G9023) for 15 min. Primary antibodies (Table 2) were prepared in universal antibody dilution (Sigma U3635) and incubated for 45 min. Following primary antibody incubation, tissue was incubated with primary antibody host species specific IgG secondary antibody, conjugated to horseradish peroxidase polymer, for 45 min. Colorization was achieved with 3,3′‐Diaminobenzidine chromogen (BD Bioscience 550,880) applied 5 min. Slides were counterstained with Gil 1 Hematoxylin (Epredia 6,765,006) as a nuclear marker, enhanced with Scott's Tap Bluing H_2_O (CM4951W). Lastly, slides were dehydrated with serial washes in 95% ethanol (1×, 2 min), 100% ethanol (2×, 2 min), and xylene (3×, 2 min) and coverslipped with permanent xylene‐soluble mounting media (Richard‐Allan Scientific™ Cytoseal™ XYL).

**TABLE 2:** Antibody information.

Primary antibody	Target	Secondary/substrate
D54D2 (1:1000; Cell Signaling)	Pan‐Aβ rabbit monoclonal antibody; labels residues Aβ‐37, Aβ‐38, Aβ‐39, Aβ‐40, and Aβ‐42	Rabbit VisUCyte™ HRP Polymer VC003‐025/3,3′‐Diaminobenzidine chromogen (BD Bioscience 550,880)
IBA1 (1:1000; Wako)	Labels myeloid derived cells in the CNS, including microglia	ImmPRESS Duet Reagent/ImmPACT DAB EqV
AT8 (1:2000; Thermo)	Phosphorylated tau at Serine 202 and Theronine 205	ImmPRESS Duet Reagent/ImmPACT Vector Red

*Note*: Description of antibody concentrations and targets.

### 2.4 Special brightfield stains

#### 2.4.1 Double chromogenic stain

Microglia and phosphorylated tau protein were visualized with a duel chromogenic stain with the ImmPRESS Duet Kit (Vector 7714) (see Table 2). Following deparaffinization and antigen retrieval tissue was quenched in BLOXALL (from Duet Kit) for 10 min, and blocked in normal horse serum for 20 min. Tissue was treated with respective primary antibodies diluted in universal antibody dilution (Sigma U3635), for 45 min, and washed in buffer. Next, the tissue was incubated in ImmPRESS Duet Reagent for 10 mins and washed with buffer (2 × 5 min). Then, slides were treated with ImmPACT DAB EqV Substrate (prepared according to Duet Kit instructions) for 5 min and washed with buffer (2 × 5 min). Slides were subsequently treated with ImmPACT Vector Red Substrate (prepared according to Duet Kit instructions) for 30 mins, washed with buffer (5 min), and washed in DiH_2_O (5 min). Lastly, slides were dehydrated and coverslipped with permanent xylene‐soluble mounting media (Richard‐Allan Scientific™ Cytoseal™ XYL).

#### 2.4.2 *N*‐Linked sialic acid staining (SNA)

To visualize *N*‐linked SA modifications in tissue, a unique biotinylated plant‐derived lectin from the Sambucas Nigra plant (SNA) was used to detect the α‐2,6 SA bond (Vector Labs Sambucus Nigra Lectin SNA, EBL‐ Biotinylated B‐1305‐2). Slides were deparaffinized with serial washes in xylene (3×, 5 min), 100% ethanol (2×, 2 min), 95% ethanol (1×, 2 min), and flushed in DiH_2_O (Leica ST5010 Autostainer XL). Heat‐induced epitope retrieval was performed by steaming slides in DiH_2_O for 30 min. After deparaffinization and antigen retrieval, tissue slides were pretreated with Protein Blocking Solution (ThermoFisher SuperBlock™ T20 37,536) for 10 min, 30% H_2_O_2_ (Fisher Chemical H325‐100) for 10 min, Streptavidin blocking (Vector Labs SP‐2002) for 15 min, Biotin blocking (Vector Labs SP‐2002) for 15 min, and Carbo‐Free Blocking Solution (Vector Labs SP‐5040) for 30 min. Then, SNA lectin was applied to the tissue at the concentration of 1.75 μg/mL (in PBS) for 30 min, followed by incubation of VECTASTAIN Elite ABC peroxidase (Vector Labs PK‐6100) for 30 min at room temperature. Colorization was achieved with 3,3′‐Diaminobenzidine chromogen (Vector Labs ImmPACT DAB SK‐4105) for 5 min. Lastly, all slides were counterstained with Gil 1 Hematoxylin (Epredia 6,765,006) as a nuclear marker, enhanced with Scott's Tap Bluing H_2_O (CM4951W). Slides were dehydrated and coverslipped with permanent xylene‐soluble mounting media.

#### 2.4.3 *O* ‐Linked SA: Periodic Acid‐Schiff–Alcian blue stain (PAS‐AB)

To visualize neutral and sulfonated *O*‐linked SA modifications, chemical based histologic stains were adapted from Dr. Yamabayashi and colleagues 1987 publication [30]. Following deparaffinization, tissue slides were incubated in freshly made 1% Periodic Acid (Fisher A223‐25) in HCl (Supelco HX0603‐4) for 30 min, rinsed in tap water for 5 min, and incubated in Schiff Reagent Hotchkiss–McManus (Sigma 6073–71) for 30 min. Then, slides were rinsed in tap water for 5 min and incubated in freshly made 0.3% Alcian blue 8GX (Sigma AS268‐10G) in 0.1 M HCl for 15 min. Lastly, slides were rinsed thoroughly in tap water and dehydrated and coverslipped with permanent xylene‐soluble mounting media.

#### 2.4.4 *O* ‐Linked SA: High iron diamine stain (HID)

To visualize sulfonated *O*‐linked SA modifications, a chemical based histologic stain was adapted from several historic publications [31, 32, 33] Following deparaffinization, tissue slides were incubated in a humidity chamber with freshly made HID solution for 18 h. To make the HID solution, 600 mL of dimethyl solution was created by combining 1.44 g of N,N dimethyl‐1,3‐phenyl‐enediamine dihydrochloride (Sigma 1,003,345,556) and 0.24 g of N,N dimethyl‐*p*‐phenyl‐enediamine dihydrochloride (Sigma 102,490,702) into 600 mL of DiH_2_O. Then, a ferric chloride stock solution was created with the combination of 60% ferric chloride solid (Sigma S826845 225) into 5% HCl. Finally, 30 mL of the ferric chloride stock solution was added to the 600 mL dimethyl solution to create the HID working solution. Following the 18‐h HID incubation, the slides were washed vigorously in tap water for at least 5 min. Lastly, slides were rinsed thoroughly in running tap water, dehydrated, and cover slipped with permanent xylene‐soluble mounting media.

### 2.5 Immunofluorescence (IF)

IF was performed to visualize *N*‐linked SA colocalization with microglia and phosphorylated tau. First, *N*‐linked SA was probed for using the aforementioned SNA protocol and Streptavidin DyLight 488 (Vector Labs SA‐5488 at 20 μg/mL in PBS). Following the SNA protocol, microglia and phosphorylated tau immunostaining was performed. Microglia were labeled with a primary antibody targeting ionized calcium binding adaptor protein 1 (IBA1, 1:1000, Wako) or plaque associated microglia (CD163, 1:50, DiH20 Steam, Novocastra) as well as phosphorylated tau (AT8, 1:2000, Thermo), respectively, for 45 min. Then, the tissue was incubated with the respective secondary antibodies, goat anti‐rabbit AlexaFluor 555 (1:500) and goat anti‐mouse AlexaFluor 647 (1:500) in the dark for 45 min. Autofluorescence artifact was blocked with incubation in Sudan Black B solution (2 min), and slides sealed with Fluoromount‐G semipermanent mounting media containing DAPI counterstain (SouthernBioTech 0100–20).

### 2.6 Image acquisition

Brightfield IHC images were acquired on the Leica Aperio AT2 Slide Scanner (version 102.0.7.5) and processed on the Aperio ImageScope Software, eSlide Manager (version 12.4.2.5010). IF images were captured on a Zeiss LSM 880 inverted confocal microscope (LSM 880 Indimo AxioObserver) using a plan Apochromat 63×/1.4 oil DIC M27 emersion objective to generate images. Images were processed using ZEN 2.3 (blue edition) software.

### 2.7 Image and statistical analyses

Brightfield whole slide images were viewed and assessed using Aperio ImageScope Software. Individual single stained positive control slides were used for optimization Color Deconvolution v9 algorithms and algorithms were used for experimental case slide series. The “tune” function was used to determine adequate color deconvolution for each histologic stain (described in supplemental methods and Figures [Link], [Link], [Link], and S5). As defined by the Aperio Imagescope operating manual and other standard analysis protocols [34, 35, 36, 37], the positive signal is defined as the pixels that are stained positive in a specified color channel. The output measurement used for analysis was the percentage of strong positive pixels in a given area or the percent area of the strong positive signal. Strong positive pixels were used because of the stringent threshold for absolute positive staining. Within a specific region of interest (ROI), the individual percent strong positive values were averaged and the measurements statistically compared were the average percent strong positive value. RO1 annotations were placed by a trained individual and ROIs were analyzed with the appropriate algorithm (see detailed protocols in Data S1). Output values were exported to Excel files for statistical analysis on Graphpad Prism 9.4.1 with Standard Deviation (SD). Statistical tests included 2‐Way Mixed Effects ANOVA with Sidak multiple comparisons, 1‐Way ANOVA, paired *t*‐test with tukey post‐hoc, and non‐paired *t*‐test.

## 3 RESULTS

### 3.1 Cross sectional analysis of AD cases

In order to study the spatial and temporal localization of both *N*‐ and *O*‐linked SA, the use of serial sections was necessary to compare regions of interest across small distances (~25 μm). To do this, 10 postmortem cases were selected from the Glenn Biggs Institute Brain Bank at UT Health Science San Antonio. These cases all had clinical history of AD, with seven cases with AD neuropathological rating, one case with intermediate AD pathology, and two cases with clinical history and limited features of AD pathology. For the purposes of this study, the eight cases with high and intermediate ADNC will be considered high pathology cases and the two cases with low ADNC will be considered low pathology cases. The variety of cases allows for the study of sialylation progression with disease aggregates (Table 1). To understand the temporal progression of AD, three brain regions were systematically chosen to investigate the spread of both Aβ plaques and tau pathology. Specially, the frontal cortex was sampled to understand regions vulnerable to early Aβ plaque deposition. Then, the hippocampus was sampled to investigate regions vulnerable to early tau pathology and convergence of Aβ and tau aggregates. Lastly, the cerebellum was sampled as both an internal control and region less vulnerable to AD pathologies. Within each brain region sampled, five serial sections were taken to probe specific cellular features and SA modifications (Figure 1A). For histologic analysis, slides from all regions were stained at the same time for consistency and quantitative digital pathology analysis. Validation of quantitative pathology techniques were thoroughly conducted on positive control tissue (Figure S1). Digital pathology analysis required the alignment of all serial sections and the placement of each ROI to be located in the same place across all five sections. The ROIs were placed in the molecular layer of the frontal cortex, CA1 and subiculum of the hippocampus, and the molecular layer of the cerebellum (Figure 1B). Utilizing this balanced neuropathological sampling approach, the investigation of location specific sialylation relative to pathological spread was achievable. With our design, we are able to qualitatively and quantitatively compare the location of SA bonds and the relative abundance or percent area of sialylation based on proximity to pathology or anatomical region. Some limitations of our study include challenges of aligning serial slides to trace a single plaque across sections. This limited the scope of plaques analyzed. Additionally, there was heterogeneity of plaque morphologies and therefore the percentage are of *O*‐SA modifications vary. This was accounted for with analyzing five regions of interest for plaque and no plaque regions in each slide across all brain regions. While it was challenging to align the sections, this is a major advantage to this study design to be able to compare SA patterns across small distances. This study clarified previously unknown tissue specific location of *N*‐ and *O*‐sialylation patterns in AD.

### 3.2 Increased *N*‐sialylation within the Aβ plaque microenvironment

**FIGURE 2:**
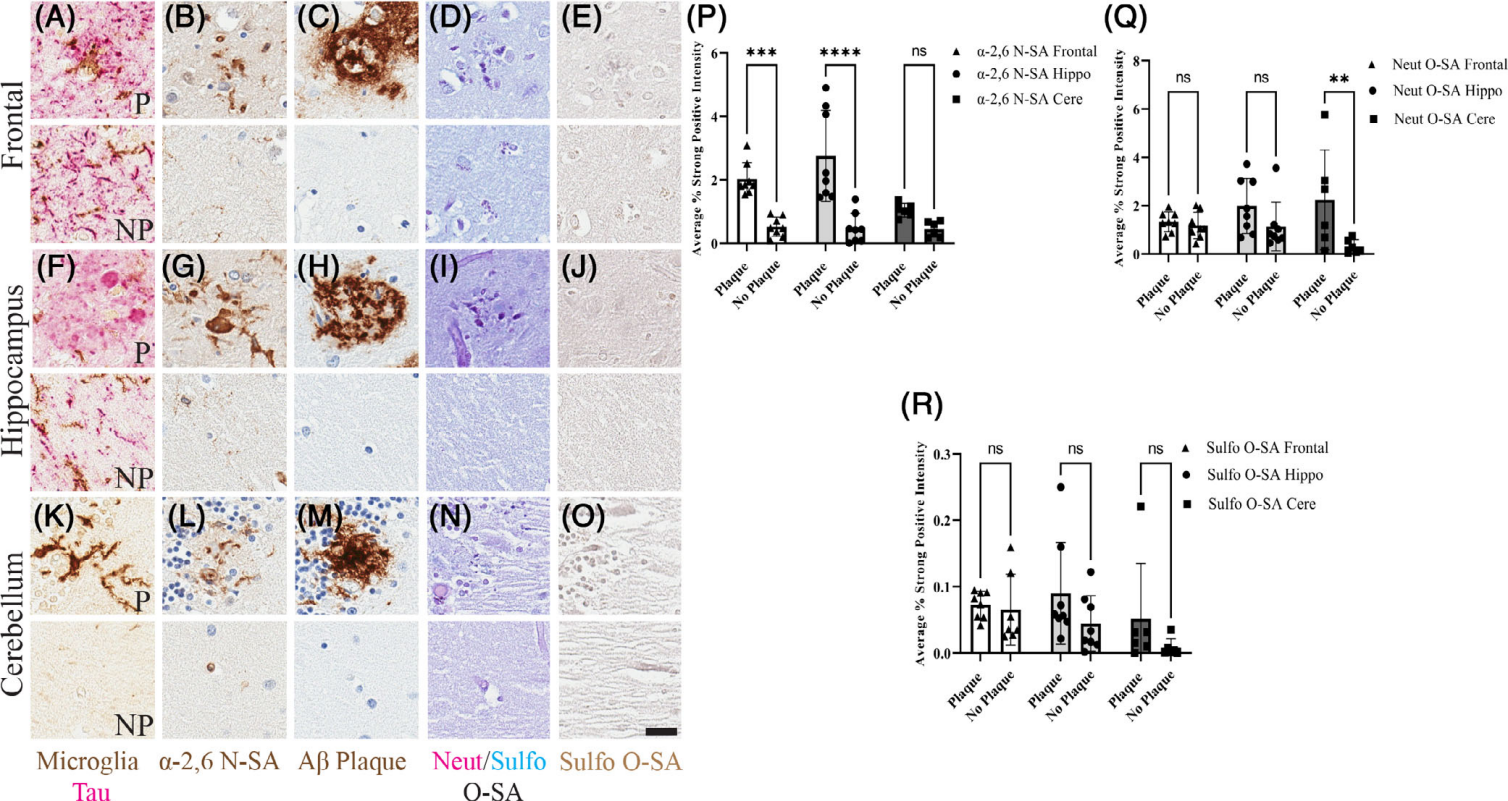
Increased sialylation in the Aβ plaque microenvironment. Significantly greater *N*‐ and *O*‐sialylation proximal to Aβ plaques. (*N* = 8). 20× images, Scale bar = 10 μm. (A) Frontal plaque (P) and no plaque (NP) ROIs labeled for phosphorylated tau and microglia. (B) Frontal P and NP ROIs labeled for α‐2,6 *N*‐SA. (C) Frontal P and NP ROIs labeled for Aβ plaque. (D) Frontal P and NP ROIs labeled for neutral/sulfonated *O*‐SA. (E) Frontal P and NP ROIs labeled for sulfonated *O*‐SA. (F) Hippocampus P and NP ROIs labeled for phosphorylated tau and microglia. (G) Hippocampus P and NP ROIs labeled for α‐2,6 *N*‐SA. (H) Hippocampus P and NP ROIs labeled for Aβ plaque. (I) Hippocampus P and NP ROIs labeled for neutral/sulfonated *O*‐SA. (J) Hippocampus P and NP ROIs labeled for sulfonated *O*‐SA. (K) Cerebellum P and NP ROIs labeled for phosphorylated tau and microglia. (L) Cerebellum P and NP ROIs labeled for α‐2,6 *N*‐SA. (M) Cerebellum P and NP ROIs labeled for Aβ plaque. (N) Cerebellum P and NP ROIs labeled for neutral/sulfonated *O*‐SA. (O) Cerebellum P and NP ROIs labeled for sulfonated *O*‐SA. (P) 2‐Way Mixed Effects ANOVA with Sidak multiple comparisons of α‐2,6 *N*‐SA average percent strong positive signal (percent area) across brain regions. (Q) 2‐Way Mixed Effects ANOVA of neutral *O*‐SA average percent area across brain regions. (R) 2‐Way Mixed Effects ANOVA of AB sulfonated *O*‐SA average percent area across brain regions. (*p* > 0.05 = NS; *p* ≤ 0.05 = *; *p* ≤ 0.01 = **; *p* ≤ 0.001 = ***; *p* ≤ 0.00 = ****).

Our previous work described the significant increase of α‐2,6 *N*‐SA within the plaque microenvironment in the 5XFAD mouse model of amyloid pathology [23]. The present study expands these findings with a holistic comparison of *N*‐ and *O*‐linked sialylation in the Aβ plaque microenvironment. To compare the levels of sialyation surrounding Aβ plaques, plaque ROIs were placed across the serial slides and the three brain regions. The plaque ROIs were compared with adjacent no plaque ROIs that did not contain Aβ pathology as internal controls. Thus, plaque and no plaque regions were paired measurements. Quantitative pathology color deconvolution algorithms were used to separate dual stained slides and measure the percent area of histologic stains. These algorithms were validated in the AD cases (Figure S2). Visually, plaque regions had greater percent area of α‐2,6 *N*‐sialylation and *O*‐sialylation compared with the no plaque regions across all brain regions (Figure 2A–O). Quantitatively, a 2‐Way Mixed Effects ANOVA with Sidak multiple comparisons of α‐2,6 *N*‐SA average percent area compared across the presence of plaque pathology and region brain region yielded a main effect of plaque pathology (*p* < 0.0001; *F* (1,19) =74.08). Additionally, there was a main effect of region (*p* = 0.0434; *F* (2,19) = 3.718), and interaction of plaque pathology × region (*p* = 0.0036; *F* (2,19) = 7.694). Within the frontal cortex, a pairwise comparison of plaque and no plaque ROIs revealed greater α‐2,6 *N*‐SA average percent area in the plaque ROI compared with no plaque (*p* = 0.0001). The same finding was present in the hippocampus (*p* < 0.0001) but not appreciable in the cerebellum (*p* = 0.2097). Thus, there was a significantly greater percent area of α‐2,6 *N*‐SA in the plaque ROIs across frontal and hippocampus regions (Figure 2P). Next, a 2‐Way Mixed Effects ANOVA with Sidak multiple comparisons for PAS positive neutral *O*‐SA average percent area yielded a main effect of pathology (*p* = 0.0012; *F* (1,19) = 14.51) and interaction of pathology × region (*p* = 0.0407; *F* (2,19) = 3.807). Pairwise comparisons demonstrated no significant difference in average percent area of neutral *O*‐SA in the frontal cortex (*p* = 0.9765) or the hippocampus (*p* = 0.1692) but significantly greater average percent area within the plaque region of the cerebellum compared with no plaque region (*p* = 0.0025) Figure 2Q). Lastly, a 2‐Way Mixed Effects ANOVA with Sidak multiple comparisons for Alcian blue‐positive sulfonated *O*‐SA average percent area yielded a main effect of pathology (*p* = 0.0220; *F* (1,19) = 6.288). There were no meaningful differences in the average percent area of sulfonated *O*‐SA in any brain region (Figure 2R). Together, these data show that there is significantly greater average percent area of *N*‐sialylation in the plaque ROIs across brain regions and less contribution of *O*‐SA in the Aβ plaque microenvironment compared with no plaque ROIs.

### 3.3 Holistic examination of N‐sialylated microglia

**FIGURE 3:**
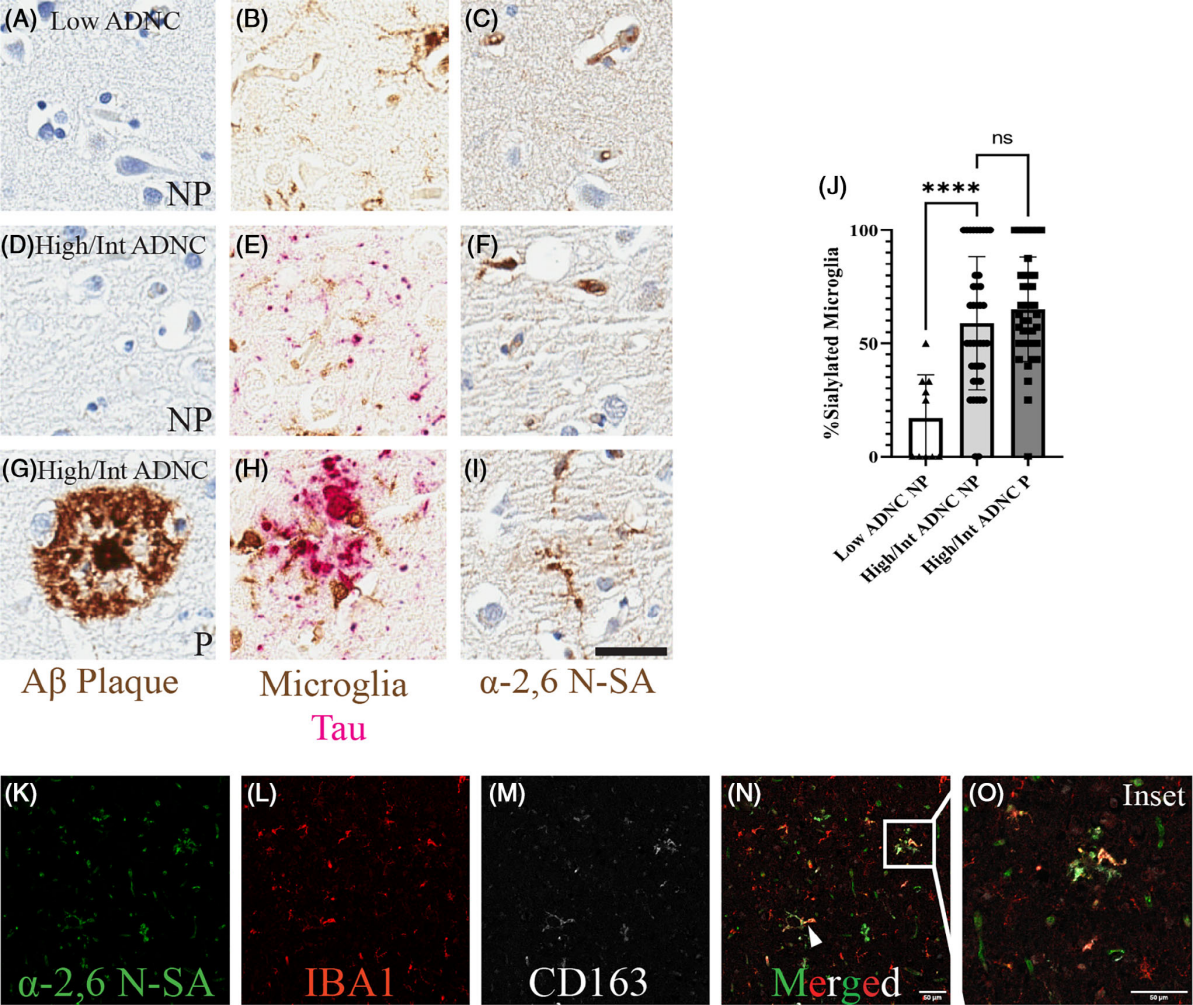
N‐sialylation differences in the frontal cortex. No differences in percent *N*‐sialylated microglia in plaque and no plaque (NP) regions, but significant differences in *N*‐SA average percent area. Low Alzheimer's disease neuropathological change (ADNC) cases (*N* = 2) and high and intermediate ADNC cases (*N* = 8). 20× brightfield images, scale bar = 25 μm. 20× immunofluorescent images, scale bar = 50 μm, inset image 2.1× zoom. (A) Low ADNC NP ROI labeled for Aβ plaques. (B) Low ADNC NP ROI labeled for phosphorylated tau and microglia. (C) Low ADNC NP ROI with α‐2,6 *N*‐SA marker. (D) High/intermediate ADNC NP ROI labeled for Aβ plaques. (E) High/intermediate ADNC NP ROI labeled for phosphorylated tau and microglia. (F) High/intermediate ADNC NP ROI with α‐2,6 *N*‐SA marker. (G) High/intermediate ADNC plaque (P) ROI labeled for Aβ plaques. (H) High/intermediate ADNC P ROI labeled for phosphorylated tau and microglia. (I) High/intermediate ADNC P ROI with α‐2,6 *N*‐SA marker. (J) 1‐Way mixed effects ANOVA with tukey post‐hoc of percent sialylated microglia across pathological burden ROIs. (K) Immunofluorescent labeling α‐2,6 *N*‐SA. (L) Immunofluorescent labeling IBA1 microglia. (M) Immunofluorescent labeling CD163 plaque associated microglia. (N) Merged panel, white arrow and box distinguish triple positive IBA1, SNA positive cells. (O) Higher magnification inset of *N* (*N* = 1) (*p* > 0.05 = NS; *p* ≤ 0.05 = *; *p* ≤ 0.01 = **; *p* ≤ 0.001 = ***; *p* ≤ 0.00 = ****).

To better understand the extent of α‐2,6 *N*‐sialylated microglia in these AD cases, we aimed to answer the question of whether all microglia are sialylated. To do so, we utilized the paired plaque and no plaque serial regions to count the number of sialylated microglia within each region. This resulted in quantifying five plaque and five no plaque regions per case. Specifically, the comparison groups were high pathology cases, defined as high and intermediate ADNC scores (*N* = 8), and low pathology cases, defined as low ADNC scores (*N* = 2). To first compare sialylated microglia in the absence of Aβ pathology, no plaque ROIs were evaluated in both low and high ADNC cases. In a 1‐Way Mixed Effects ANOVA with tukey post‐hoc comparing percent sialylated microglia within the no plaque ROIs, there was a significant decrease (*p* < 0.0001; *F* (2,8) = 18.59) in microglia sialylation in the low ADNC no plaque ROIs compared with the high ADNC no plaque ROIs (Figure 3A–F,J). In the presence of Aβ pathology, plaque and no plaque ROIs were compared within the high ADNC cases. There was no significant difference in the percentage of sialylated microglia within the plaque ROIs and no plaque ROIs (*p* = 0.4516; *F* (2,8) = 18.59) (Figure 3D–I,J). Interestingly, not all microglia are sialylated in the plaque microenvironment of high ADNC cases, as approximately 65% of microglia are sialylated in the plaque ROIs (Figure 3J). This comparison does not account for the percent area of α‐2,6 *N*‐sialylation but simply the presence of sialylated microglia. As described in Figure 2P, the positive percent area of α‐2,6 *N*‐sialylation is significantly greater in plaque ROIs compared with no plaque ROIs. Thus, this data supports a significantly greater percent sialylated microglia in high AD neuropathological change cases compared with low AD neuropathological change cases. Additionally, there is evidence that suggests not all microglia are sialylated, but there is an Aβ pathology specific increase in α‐2,6 *N*‐sialylation percent area within the plaque microenvironment. To localize *N*‐sialylation to disease relevant microglia populations, co‐immunofluorescence was used to label plaque associated microglia with the CD163 antibody (Figure 3K–N). In the merged image (Figure 3N), the white arrow and box highlight the overlapping fluorescent signal of α‐2,6 SA, IBA1, and CD163. A higher magnification view of the inset image allows us to visualize the α‐2,6 *N*‐sialyation of CD163 positive plaque associated microglia (Figure 3O). Through this investigation, we provide additional evidence for increased α‐2,6 *N*‐SA on plaque associated microglia subpopulations.

### 3.4 Sialylation patterns within cored and diffuse plaque morphologies

**FIGURE 4:**
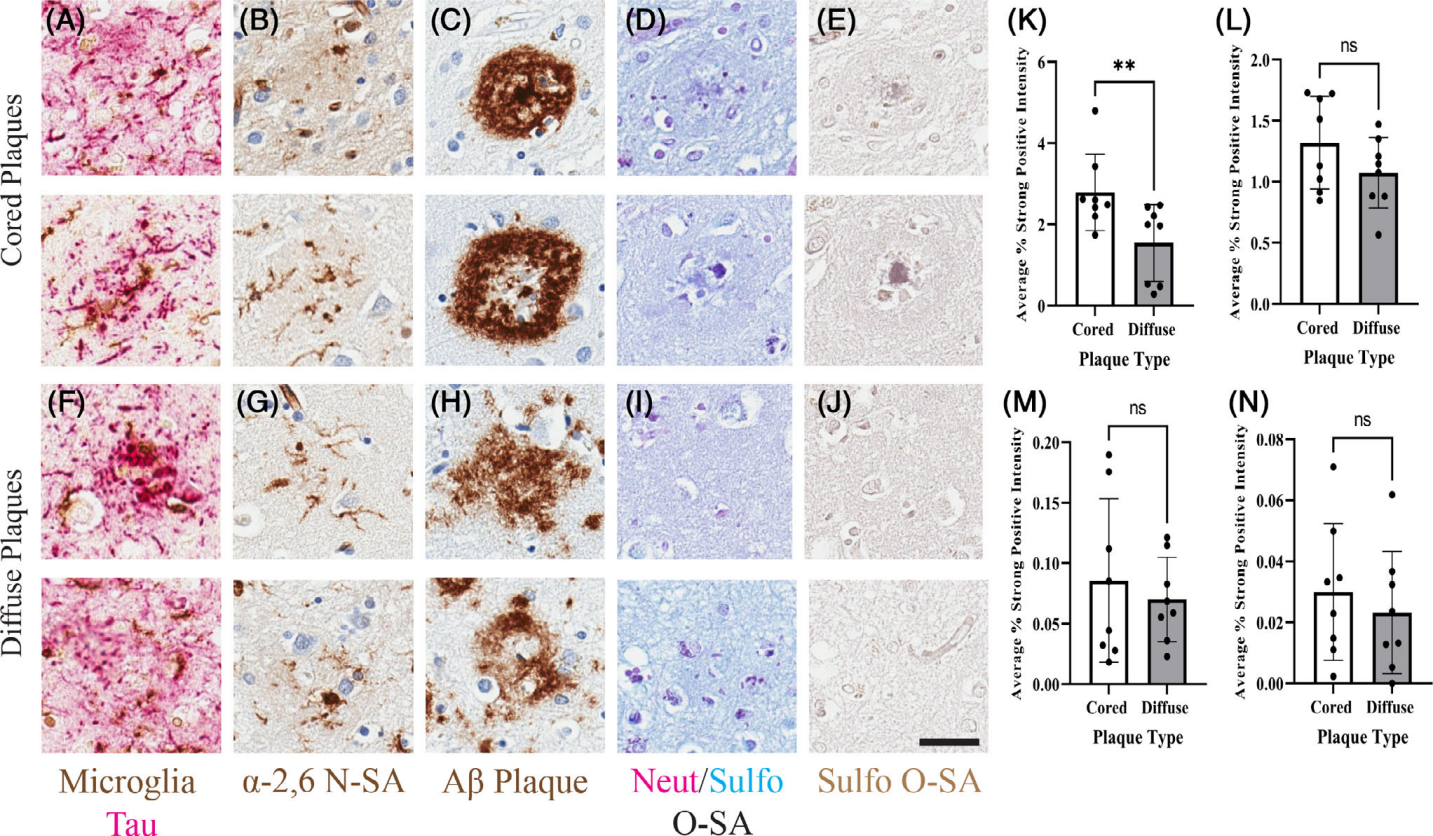
Limited differences in sialylation between cored and diffuse Aβ plaques. Increased N‐sialylation in both cored and diffuse plaques regions. No significant difference in molecular and SA markers when comparing cored and diffuse plaque microenvironments. (*N* = 8). 20× images, scale bar = 25 μm. (A) Frontal cored plaque ROIs (2×) labeled for phosphorylated tau and microglia. (B) Frontal cored plaque ROIs (2×) labeled for α‐2,6 *N*‐SA. (C) Frontal cored plaque ROIs (2×) labeled for Aβ plaque. (D) Frontal cored plaque ROIs (2×) labeled for neutral/sulfonated *O*‐SA. (E) Frontal cored plaque ROIs (2×) labeled for sulfonated *O*‐SA. (F) Frontal diffuse plaque ROIs (2×) labeled for phosphorylated tau and microglia. (G) Frontal diffuse plaque ROIs (2×) labeled for α‐2,6 *N*‐SA. (H) Frontal diffuse plaque ROIs (2×) labeled for Aβ plaque. (I) Frontal diffuse plaque ROIs (2×) labeled for neutral/sulfonated *O*‐SA. (J) Frontal diffuse plaque ROIs (2×) labeled for sulfonated *O*‐SA. (K) Paired parametric *t*‐test of average percent area of α‐2,6 *N*‐SA. (L) Paired parametric *t*‐test of average percent area of neutral *O*‐SA. (M) Paired parametric *t*‐test of average percent area of sulfonated *O*‐SA, measured with AB. (N) Paired parametric *t*‐test of average percent area of sulfonated *O*‐SA, measured with HID (*p* > 0.05 = NS; *p* ≤ 0.05 = *; *p* ≤ 0.01 = **; *p* ≤ 0.001 = ***; *p* ≤ 0.00 = ****).

To better understand the contribution of sialyation based on Aβ pathology, the evaluation of sialylation patterns of cored and diffuse Aβ plaque pathology was performed. In this analysis, the contribution of *O*‐linked SA was of particular interest based on the previous studies of increased *O*‐linked SA on proteins associated with Aβ plaques and potential regulatory effect of O‐sialylation on tau hyperphosphorylation [22, 38, 39]. To complete this analysis, cored and diffuse plaque regions in the frontal cortex of high pathology cases were compared with tease apart sialylation contributions in each plaque morphology. Qualitatively, two cored plaque paired regions (Figure 4A–E) had classical cored plaque morphologies with visible *O*‐SA features. In contrast, two diffuse plaque paired Figure 4F–J) had dispersed amyloid staining and less intense *O*‐SA levels. Quantitative differences were assessed with paired parametric T‐tests comparing cored and diffuse ROIs sialylation intensities. Cored plaque ROIs had significantly greater α‐2,6 *N*‐SA average percent area compared with diffuse plaque ROIs (*p* = 0.0028) (Figure 4K). For *O*‐SA, there was no significant difference between cored and diffuse plaque ROIs in average percent area for PAS positive neutral *O*‐SA (*p* = 0.0763), Alcian blue‐positive sulfonated *O*‐SA (*p* = 0.2750), and HID positive sulfonated *O*‐SA (*p* = 0.5520) (Figure 4L–N). Taken together, Aβ plaque microenvironments are *N*‐ and *O*‐sialylated regardless of plaque morphology, with greatest contribution from α‐2,6 *N*‐SA in our cases.

### 3.5 Increased *N*‐sialylation of microglia relative to tau pathology

**FIGURE 5:**
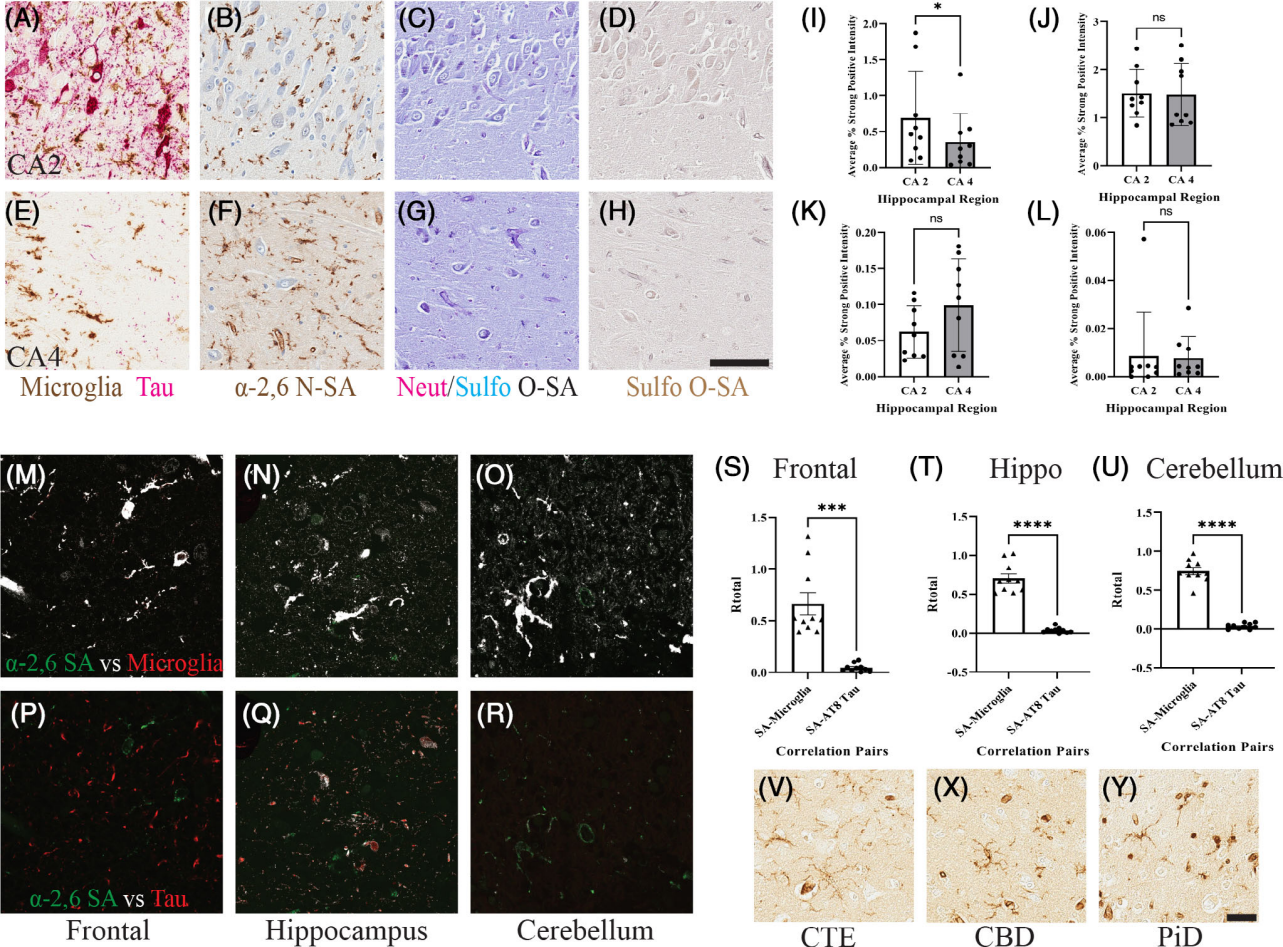
Increased sialylation not driven by tau pathology in AD cases. Minor increase of sialylation relative to phosphorylated tau pathology. CA2 region of the hippocampus defined as a high tau burden region and CA4 as a low tau burden region (*N* = 9). 20× images, Scale bar = 25 μm. (A) CA2 ROI labeled for phosphorylated tau and microglia. (B) CA2 ROI labeled for α‐2,6 *N*‐SA. (C) CA2 ROI labeled for neutral and sulfonated *O*‐SA. (D) CA2 ROI labeled for only sulfonated *O*‐SA. (E) CA4 ROI labeled for phosphorylated tau and microglia. (F) CA4 ROI labeled for α‐2,6 *N*‐SA. (G) CA4 ROI labeled for neutral and sulfonated *O*‐SA. (H) CA4 ROI labeled for only sulfonated *O*‐SA respective makers. (I) Paired *t*‐test of average percent area for α‐2,6 *N*‐SA between CA2 and CA4. (J) Paired *t*‐test of average percent area for neutral *O*‐SA between CA2 and CA4. (K) Paired *t*‐test of average percent area for sulfonated *O*‐SA, measured with AB, between CA2 and CA4. (L) Paired *t*‐test of average percent area for sulfonated *O*‐SA, measured with HID, between CA2 and CA4. (M) Frontal cortex α‐2,6 *N*‐SA (green) and microglia (red) with a pixel‐to‐pixel Pearson correlation analysis, positively correlated pixels in white. (N) Hippocampus α‐2,6 *N*‐SA (green) and microglia (red), positively correlated pixels in white. (O) Cerebellum α‐2,6 *N*‐SA (green) and microglia (red), positively correlated pixels in white. (P) Frontal cortex α‐2,6 *N*‐SA (green) and phosphorylated tau (red). (Q) Hippocampus α‐2,6 *N*‐SA (green) and phosphorylated tau (red). (R) Cerebellum α‐2,6 *N*‐SA (green) and phosphorylated tau (red). (S) Paired *t*‐test comparison of Pearson correlation Rtotal in the frontal cortex. (T) Paired *t*‐test comparison of Pearson correlation Rtotal in the hippocampus. (U) Paired *t*‐test comparison of Pearson correlation Rtotal in the cerebellum. (V) α‐2,6 *N*‐sialylation in CTE (insula). (X) α‐2,6 *N*‐sialylation in CBD (frontal). (Y) α‐2,6 *N*‐sialylation in PiD (frontal) (*p* > 0.05 = NS; *p* ≤ 0.05 = *; *p* ≤ 0.01 = **; *p* ≤ 0.001 = ***; *p* ≤ 0.00 = ****).

With thorough investigation into sialylation and Aβ plaques, the association between SA and phosphorylated tau was unknown. Thus, a multipronged approach was taken to investigate the correlation of *N*‐ and *O*‐linked SA relative to tau pathology. First, within the hippocampus of the serial sections, two regions were identified, the CA2 region of high tau pathology with little Aβ pathology (Figure 5A–D) and CA4 region of low tau pathology (Figure 5E–H). Then, the 9 cases with notable tau pathology were compared with respective CA2 and CA4 ROIs. Qualitatively, the two hippocampal regions had noticeable differences including the presences of NFT pathology in the CA2 (Figure 5A,E). Quantitative differences between the forms of sialylation were compared between the regions with a paired parametric *t*‐test. Interestingly, there was a significantly higher average percent area of α‐2,6 *N*‐SA in the CA2 regions of high tau pathology compared with the CA4 (*p* = 0.0431; Figure 5I). Then, comparing the average percent area of the other *O*‐SA markers, there were no significant differences for neutral (*p* = 0.8815), sulfonated measured with Alcian blue (*p* = 0.1366), and sulfonated measured with HID (*p* = 0.8196; Figure 5J–L). While this data supports a difference in α‐2,6 *N*‐SA percent area, sialylation of tau pathology is still unknown. To answer this question, colocalization analysis was used to compare the correlation of phosphorylated tau and α‐2,6 *N*‐SA. Non‐serial sections from the 10 cases were evaluated using multicolor immunofluorescence to probe α‐2,6 *N*‐SA, phosphorylated tau, and microglia. In order to localize α‐2,6 *N*‐SA, the pixel‐to‐pixel correlation of the α‐2,6 *N*‐SA channel and phosphorylated tau or microglia channel were calculated respectively with a Pearson correlation. Quantitative differences between the Pearson correlation coefficient (*R*) values were determined with a paired parametric *t*‐test for each brain region. α‐2,6 *N*‐SA was positively correlated with microglia and not phosphorylated tau (*p* = 0.0002; Figure 5S). This is visually appreciable when positively correlated pixels of α‐2,6 *N*‐SA versus microglia are depicted in white (Figure 5M) and lack of correlated pixels results in no white coloration (α‐2,6 *N*‐SA vs. phosphorylated tau) (Figure 5P). The same comparison was made in the hippocampus with strong positive correlation of α‐2,6 *N*‐SA and microglia (*p* < 0.0001) and not with phosphorylated tau (Figure 5N,Q,T). Lastly, the comparison within the cerebellum was made and replicated the significant correlation of α‐2,6 *N*‐SA and microglia (*p* < 0.0001) without localization on phosphorylated tau (Figure 5O,P,U). Together, this data supports the finding that sialylation does not appear to be strongly associated with tau pathology in AD. While we see significant α‐2,6 *N*‐sialylation in AD independent of tau pathology, α‐2,6 *N*‐sialylation is apparent in other tauopathies: chronic traumatic encephalopathy (CTE), corticobasal degeneration (CBD), and Pick's disease (PiD) all have α‐2,6 sialylated microglia (Figure 5V–Y). These data suggest that there may be tauopathy‐specific features that enhance α‐2,6 *N*‐SA in AD and these other tauopathies that are less appreciable without the presence of amyloid pathology that strongly drives α‐2,6 *N*‐SA expression.

## 4 DISCUSSION

The goal of the current study was to (1) characterize histology techniques to visualization the sialylation landscape in human brain tissue; (2) determine where specific forms of *N*‐ and *O*‐linked SA modifications are located in AD brains relative to Aβ plaques, microglia, and phosphorylated tau pathology; and (3) utilize brain regions vulnerable to AD pathology to better determine spatiotemporal distribution of sialylation patterns.

**FIGURE 6:**
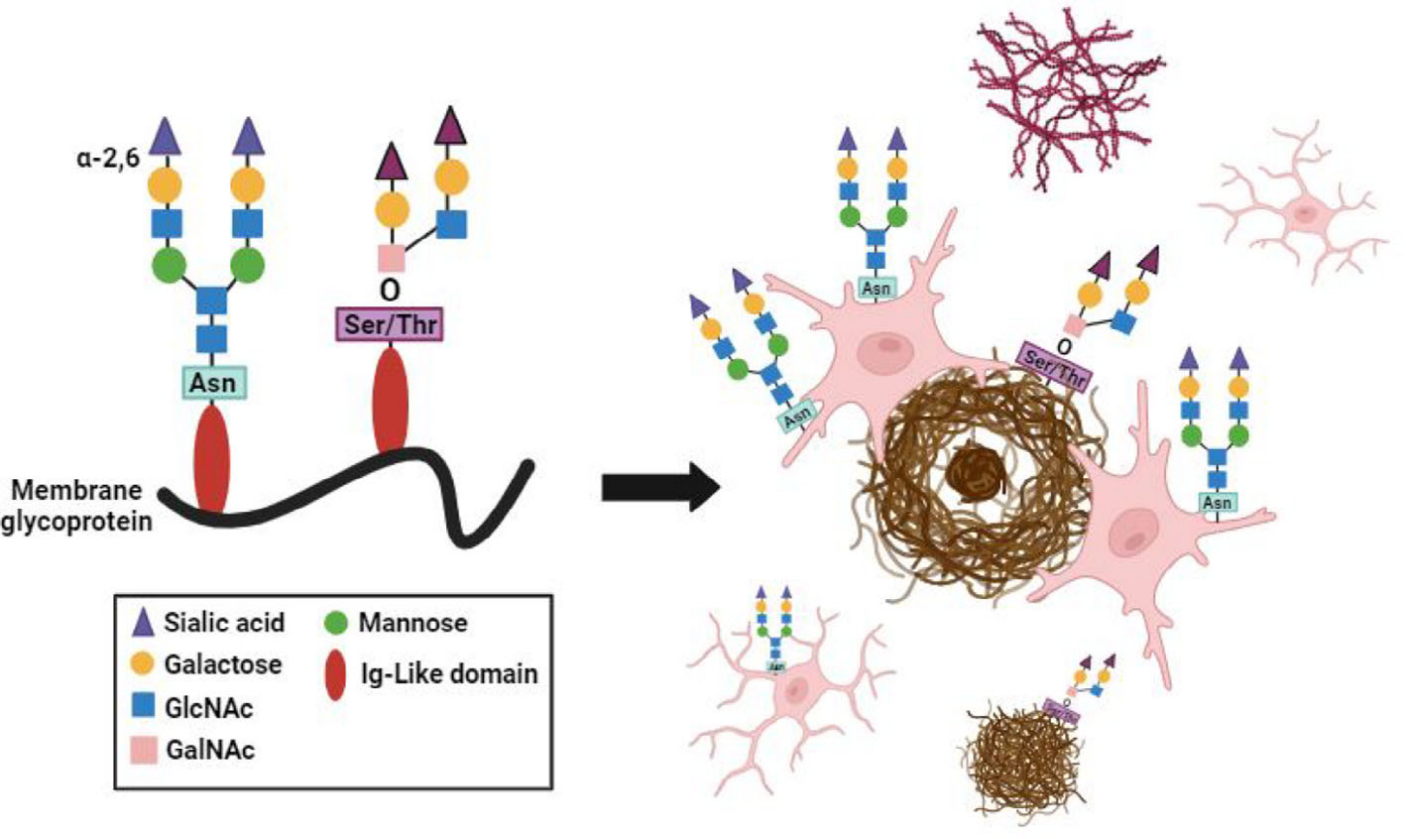
Cellular and aggregate specific representation of sialylation landscape in AD.Graphical summation of sialylation localization relative to plaque pathology and tau pathology.

Histological techniques to probe glycosylation have not been widely used in either brain tissue or AD cases. Glycosylation is the most common form of post‐translational modification (PTM) with a projected 50% of proteins, as well as lipids, have these modifications that contribute to various cellular activities [14, 15, 40, 41]. Glycans serve many functions including cellular recognition with modulation of membrane receptor signaling via spatial and steric modifications, membrane organization with bulk charge differences, immune regulation through immune escape strategies and receptor‐mediated activity changes, and regulation of protein trafficking with *N*‐ and *O*‐glycosylation at the Golgi [42, 43, 44, 45]. Many glycans include the addition of terminal SA or neuraminic acid through a process of sialylation. There are two main forms of protein sialylation, *N*‐linked and *O*‐linked (Figure 6). The main difference between these forms of sialylation includes the structure of the glycoconjugate and location of synthesis. *N*‐sialylation is the addition of *N*‐acetylneuraminic acids (Neu5Ac) to a nitrogen group on asparagine amino acids. *N*‐glycosylation modifications share a common core structure and SA are added by sialyltransferase enzymes in the Golgi apparatus. In contrast, *O*‐glycosylation modifications have diverse core structures. These glycans are covalently bound to hydroxyl oxygen groups on serine or threonine amino acids with additional modification of SA by glycosyltransferase enzymes and can be further modified with other PTMs such as acylation and sulfonation [46]. Prior to the present study, visualization of these glycan modifications within the brain has been limited. To visualize α‐2,6 *N*‐SA, we used the plant‐based lectin SNA which specifically labels this modification (Figure S2). However, there are few stains available to distinguish *O*‐linked SA modifications. *O*‐sialylation has predominately been investigated in the context of mucins, or glycosylated proteins produced by epithelial tissue, and vascular endothelial cells [47]. In 1964 Spicer and others discovered the labeling of sulfonated PTM in a variety of mucosal tissues with HID staining [48]. The specificity of these modifications was elucidated by Voltz, Reid, and others to define the labeling of neutral and sulfate ester groups of SA modifications with the combination of HID and Alcian blue procedures in colon and intestinal tissues [33]. In the present study, the goal was to optimize histological stains previously used in other organs to effectively label *O*‐linked SA in the brain. We were able to create HID and Alcian blue histological stains in AD brain tissue and validate the labeling of *O*‐SA. This is an advance over previous studies that used mass spectrometry of CSF and found bond‐specific increases in *N*‐ and *O*‐glycans in AD brains compared with controls [18, 19]. *N*‐glycoproteomic analysis of human AD brains found dysregulated *N*‐glycosylation associated with extracellular matrix dysfunction, neuroinflammation, synaptic dysfunction, cell adhesion alteration, lysosomal dysfunction, endocytic trafficking dysregulation, endoplasmic reticulum dysfunction, and cell signaling dysregulation [20]. Recent advances in brain matrix‐assisted lasor desorption/ionization mass spectrometry imaging (MALDI‐MSI) offer the promise of regional and subcellular resolution [49] that will further advance our understanding of the spatial organization of the glycome in AD. Early MSI studies in the AD dentate gyrus found disordered localization of the glycolipid ganglioside GM1 [50], while a recent study found hyper *N*‐glycosylation within the frontal cortex in the 5XFAD mouse model of amyloid pathology and rTg4510 mouse model of tau pathology. Alternatively, when comparing levels of *N*‐glycosylation in post‐mortem brain tissue of healthy control versus AD cases, there were significant differences between cognitively normal and AD cases with respect to hyper *N*‐glycosylation within the frontal cortex grey matter [51]. However, this study could not measure sialylation in their samples. Other techniques are evolving that could be used to identify bond‐specific changes in AD, such as isomer‐targeted derivatization strategy to identify SA bonds in tissues, biofluids, and cultured cells, allowing for the identification of several *N*‐sialylation bonds including α‐2,3 and α‐2,8 but not α‐2,6 SA bonds [52]. To our knowledge our quantitative approach to localizing *N*‐ and *O*‐linked sialylation modifications histologically alongside other pathological features is a novel approach in the context of post‐mortem AD brain tissue.

Recently, Nosova and others reported labeling of Alcian blue‐positive glycoproteins within Aβ plaques in a mouse model of AD [53]. This finding aligns with our results of Alcian blue positivity in the Aβ plaque environment of post‐mortem AD brain tissue. Specifically, we found that there was significantly increased neutral and sulfonated *O*‐SA localized to plaques themselves. Our data captured percent area of positive histological signal across slides, measuring variation of percent area of *O*‐SA staining of Aβ plaques (Figure S6). Ultimately, Aβ plaques appear to be decorated with varying levels of *O*‐SA. To understand the variation in *O*‐SA percent area near amyloid plaques, we examined cored and diffuse plaques separately. There were no major differences in the *O*‐sialylation landscape when comparing classical cored plaque and diffuse plaque morphologies (Figure 4L–N). This finding is intriguing because *O*‐sialylated glycoproteins play an important role in cell–cell adhesion and binding of ligands [47]. *O*‐glycosylation has been observed on the proteins clusterin and apolipoprotein E (APOE) within the Aβ plaque [54, 55, 56]. There are no comparable analysis of glycosylation and sialylation enzyme activity among APOE isoforms. But, sialylation of APOE is likely critical for proper APOE‐HDL association. Thus, one mechanism for APOE4 exacerbation of amyloid pathology in AD is caused by the decreased sialylation of APOE4 leading to issues with lipidation and ultimately preference for VLDL, reduced binding affinity inducing issues with amyloid beta clearance [57, 58]. Additional *O*‐glycosylation has been shown to disrupt amyloid precursor protein (APP) processing by reducing Aβ1‐40 generation with marginal impact on Aβ1‐42 generation, potentially because of *O*‐glycosylation disrupting protein localization and trafficking, in turn indicating excessive APP *O*‐glycosylation alters processing by secretases [59]. Overall, these findings align with increased *O*‐sialylation of proteins associated with Aβ plaques.

We previously found that plaque‐associated microglia had increased α‐2,6 *N*‐SA in the 5XFAD mouse models of amyloid pathology that was not observed [23]. Aligning with our past findings, here we observed increased α‐2,6 *N*‐SA on microglia in the Aβ plaque microenvironment compared with no plaque regions (Figure 2A–O). We found ~65% of microglia are α‐2,6 *N*‐sialylated in the plaque environment of the middle frontal gyrus (Figure 3J). Cases with high and intermediate ADNC had significantly increased microglia α‐2,6 N‐sialylation within the no plaque regions compared with low ADNC no plaque regions (Figure 3J). Within the high ADNC cases, there was no difference in the number of α‐2,6 N‐sialylated microglia in the plaque and no plaque regions (Figure 3J). This suggests that the same proportion of microglia are α‐2,6 N‐sialylated in high pathology AD cases regardless of the presence of plaques in the immediate microenvironment. However, the percent area of α‐2,6 *N*‐SA is significantly higher near plaques compared with adjacent regions without plaques (Figure 2P). Microglia are more intensely α‐2,6 *N*‐sialylated near cored plaques (Figure 4K), suggesting that α‐2,6 *N*‐sialylated microglia may aid in the compaction of plaques [60, 61]. Taken together, these data suggest that α‐2,6 *N*‐sialylated microglia increase during AD, especially around plaques. These data align with *N*‐glycoproteomic data showing that hyperglycosylation [20].To better understand the phenotype of α‐2,6 *N*‐sialylated microglia, we stained for both α‐2,6 *N*‐SA and CD163, a marker for perivascular macrophages and plaque‐associated [62, 63]. We found that CD163 positive plaque‐associated microglia are highly α‐2,6 *N*‐sialylated (Figure 3K–O). Interestingly, not all IBA1 positive microglia are CD163 positive, highlighting a distinguishment of this marker for a potentially disease relevant microglia subpopulation. In addition, it appears that microglia with increased soma size morphology are CD163 positive sialylated microglia while more ramified microglia are only moderately α‐2,6 *N*‐sialylated (Figure 3N,O). Together, our data aligns with previous findings of CD163 as a plaque‐associated microglia marker in AD brains and supports our hypothesis that microglial α‐2,6 *N*‐sialylation is associated with a distinct subtype of microglia in AD. This may be functionally significant to the role of microglia in plaque compaction and other immune functions. Microglia utilize cell surface expression of SA as an immune checkpoint as these modifications act as ligands for SA‐binding immunoglobulin‐like lectins (Siglecs) [41, 64, 65]. The removal of *N*‐linked SA by neuraminidase enzymes in a process called desialylation can enhance microglia phagocytosis [8, 66]. Importantly, changes in microglia glycosylation may be in part because of the activation of microglia response to stimuli. Immortalized BV2 microglia stimulated with LPS, fibrillar amyloid beta, and tau respond with increased desialylation of the cell surface SA residues and in turn increase phagocytic activity [66]. Additionally, proinflammatory cytokine expression is increased when human IPSC microglia are exposed to LPS and oligomeric amyloid beta leading to comparable increases in specific forms of glycosylation including fucosylation of glycoconjugates [67]. Conversely, specific modifications of l‐fucosylation of glycans on the cell surface of microglia attenuate LPS stimulation of proinflammatory cytokine release [68]. Thus, there are unique changes in microglia glycosylation states based on stimuli and model, highlighting the need to better understand specific changes in SA function within in vivo models.

There are numerous candidate microglial proteins that may show enhanced α‐2,6 *N*‐sialylation during AD. One intriguing candidate protein is TNFR1, whereby α‐2,6 *N*‐sialylation reportedly enhances pro‐inflammatory signaling on microglia [69]. Conversely, glycosylation of the microglia receptor CD200R is important for maintaining microglia in a homeostatic state upon interaction with neuronal CD200 [70] and *N*‐glycosylation of peroxisome proliferator‐activated receptor γ suppresses microglia activation [71]. Several microglia‐associated *N*‐glycoproteins are hyperglycosylated in AD, including Stabilin‐1, Intercellular Adhesion Molecule 1 (ICAM‐1), the NADPH oxidase (NOX2) component cytochrome b‐245, clusterin [20].Taken as a whole, our data supports an increase in *N*‐sialylation may potentially modulate microglia functions within the plaque environment.

We also investigated the spatial relationship between tau pathology and sialylation. Early work found that glycosylation modification of tau may contribute to downstream hyperphosphorylation and NFT development, specifically *N*‐linked SA modifications [72]. Tau can also be *O*‐sialylated which may contribute to regulation of tau phosphorylation: as paired helical filament (PHF) tau is not *O*‐sialylated, a hypothesis arose that PHF tau may be a result of impaired *O*‐sialylation and leads to further hyperphosphorylation of tau [73]. In our cases, there was a significant increase in α‐2,6 *N‐*SA in the CA2 region of the hippocampus, a region for moderate tau pathology, compared with the CA4 region, a region typically spared from tau pathology (Figure 5A–F,I). Interestingly, there was no significant difference in any form of *O*‐sialylation comparing two regions differentially impacted by tau pathology (Figure 5C–H,J–L). While there is a significant increase in *N‐*SA in the CA2 region of the hippocampus, the question of whether tau pathology was sialylated itself. Our data supports no positive correlation between *N*‐SA and phosphorylated tau (Figure 5P–U). However, there is a positive correlation between *N*‐SA and microglia, supporting the specificity of α‐2,6 *N‐*SA on microglia cell surface (Figure 5M–O,S–U), replicating our previous findings within the plaque microenvironment [23]. Intriguingly, we found that microglia in other tauopathies exhibit α‐2,6 *N*‐sialylation (Figure 5V–Y). This suggests AD and tauopathies share this phenotype to increase *N*‐SA, but within AD this may be partially more driven by amyloid pathology and through differing pathways for tauopathies. Our findings align with previously reported single nuclei RNA sequencing of human AD microglia, identifying distinct differences of microglia RNA expression with the presence of only Aβ plaque pathology compared with both Aβ and tau pathology [74]. Together, this supports there are microglia subtypes interacting with Aβ and tau pathology in AD and increased α‐2,6 *N‐*SA may play a larger role in the microglia interacting with Aβ plaque pathology.

Pathological characterization of AD includes the staging of pathology according to Aβ plaques with Thal phase criteria, tau aggregates with Braak staging, and semiquantitative scoring of neuritic plaques with according to the CERAD [27]. Each pathology staging criteria accounts for regional distribution of aggregates, allowing for the understanding of differential pathological distribution. In our study design, we sampled three brain regions, frontal cortex, hippocampus, and cerebellum, known to harbor various levels of AD pathological burden at different stages of disease (Table 1). This allowed for the investigation of regions particularly vulnerable to both Aβ and tau pathology to better understand sialylation percent area relative to temporal pathological deposition. Early Aβ pathology deposition was observed in the frontal cortex with some tau pathology, early tau pathology deposition was observed in the hippocampus as well as the convergence of later phase Aβ pathology deposition, and extremely late stage Aβ pathology was observed in the cerebellum. Comparing *N*‐sialylation across brain regions, there was significantly greater α‐2,6 *N*‐SA in the frontal cortex and hippocampus compared with the cerebellum, but there was no significant difference between the frontal and hippocampus plaque regions (Figure 2P). While we expected the greatest α‐2,6 sialylation in the frontal cortex because this region has Aβ plaque deposition earliest and potentially longer accumulation time for *N*‐SA, the lack of difference between frontal and hippocampal regions may potentially indicate hippocampal microglia display alterations in sialylation patterns because of convergence of pathologies in this region of the brain. While it appears Aβ pathology strongly drives α‐2,6 *N*‐SA percent area and tau pathology influences sialylation to a much lesser degree, the limited time of Aβ pathology deposition in the hippocampus may not be substantial enough to lead to significantly higher α‐2,6 *N*‐SA positive pixel levels as expected. When comparing neutral *O*‐sialylation across regions, there was a significant main effect of amyloid pathology and interaction between amyloid pathology and region. But there were no specific increases in this modification based on brain region alone (Figure 2Q). This indicates that the presence of amyloid pathology does increase neutral *O*‐sialylation percent area but not in a regional manner, potentially suggesting *O*‐SA levels are not tied to regional distribution of pathology as originally hypothesized. Similarly, there was no difference in sulfonated *O*‐SA based on brain region. Therefore, sulfonated *O*‐SA levels are not related to pathological distribution across brain regions. Altogether, this data aligns with the original hypothesis that *N*‐sialylation of microglia is more strongly associated with brain regions vulnerable to Aβ pathology and *O*‐sialylation of proteins potentially within the Aβ plaque microenvironment are not associated with the temporal progression of region impacted my AD pathology in these cases.

Our study utilized postmortem human tissue and limits our ability to probe active molecular mechanisms behind the increases in sialylation that we observed. In our previously published work, we found that neuraminidase 1 (Neu1), the enzyme that cleaves α‐2,6 *N*‐SA, expression is decreased and St6gal1, the sialyltransferase enzyme that deposits α‐2,6 *N*‐SA, expression is increased in an age‐dependent manner in 5XFAD mice which we posit could mediate the increase of *N*‐SA we observed [23]. Data from humans suggest there is no significant baseline correlation of Neu1 RNA expression with Braak or CERAD scores in AD brains. Additionally, there is no correlation of St6gal1 RNA expression with AD pathology [75]. Therefore, this suggests there is no association between RNA expression of enzymes responsible for α‐2,6 *N*‐SA levels in AD but this does not account for protein expression. While our data supports increased α‐2,6 *N*‐SA in the context of AD pathology, we are probing levels histologically and future quantitative protein measurements would shed light on these expression differences. We propose our findings of increased α‐2,6 *N*‐SA percent area in AD cases is a product of disease and microglia specific changes throughout the pathological progression of the disease and warrant continued investigation into intermediate enzymes along the sialylation pathway.

## 5 CONCLUSION

Previous studies have highlighted dysregulation in the glycome during AD. This project uses novel histological methods to localize changes in glycosylation relative to pathological, cellular, and anatomical features in human AD. We found significant increases in α‐2,6 *N*‐sialylation on plaque associated microglia and in neutral and sulfonated *O*‐SA on Aβ plaques (Figure 6). Additionally, our data suggest that α‐2,6 N‐sialylated microglia may represent a novel subset of neurodegenerative microglia that may be functionally significant. The sialylation landscape across plaque morphologies demonstrates increased *N*‐sialylation of microglia surrounding cored plaques compared with diffuse plaques. However, there is significantly higher α‐2,6 *N*‐SA compared with *O*‐SA in the plaque microenvironment. In AD cases, increased neither *N*‐ nor *O*‐sialylation appears to be related to phosphorylated tau pathology, but *N*‐sialylation of microglia is present in other types of tauopathy. Overall, our findings encourage further investigation into the functional consequences of each form of sialylation.

## AUTHOR CONTRIBUTIONS

*Study conception and design*: Caitlyn Fastenau, Sarah C. Hopp, and Kevin F. Bieniek. *Material preparation, data collection and analysis*: Caitlyn Fastenau, Madison Bunce, and Mallory Keating. All authors read and approved the final manuscript.

## FUNDING INFORMATION

This work was supported by the National Institutes of Health [T32AG021890 to CF, R21AG072423 and pilot funding under P30AG013319 to SCH, and P30AG066546 to KFB]; the Texas Alzheimer's Research and Care Consortium to KFB, and the Bartell and Mollie Zachry Endowment for Alzheimer's Research and Patient Care to KFB.

## CONFLICT OF INTEREST STATEMENT

Authors have no conflicts of interest.

## ETHICS STATEMENT

All activities herein were reviewed the UT Health Science Center San Antonio Institutional Review Board and deemed exempt non‐human subject research.

## Supporting information


**Data S1.** Supporting Information.

**Data S2.** Supporting Information.

**Data S3.** Supporting Information.

**Data S4.** Supporting Information.

**Data S5.** Supporting Information.

**Data S6.** Supporting Information.

**Data S7.** Supporting Information.

**Data S8.** Supporting Information.

**Data S9.** Supporting Information.

**Data S10.** Supporting Information.

**Data S11.** Supporting Information.

**Supplementary Figure S1.** Imagescope algorithm validation on positive control tissue.
Systematic validation of O‐ and N‐SA makers utilizing Imagescope color deconvolution (CD) algorithm. 20x images, Scale bar = 25 μm. (A) Representative image of brightfield PAS only stain. (B) Representative image of brightfield AB only stain. (C) Representative image of brightfield PAS‐AB combo stain. (D) Representative image of PAS‐stained tissue analyzed with AB CD algorithm, no color represents no PAS signal is being identified with the AB algorithm. (E) Representative image of AB‐stained tissue analyzed with AB CD algorithm, expectation of adequately deconvoluted blue color is achieved. (F) Representative image of PAS‐AB stained tissue analyzed with AB CD algorithm, expectation of adequately deconvoluted blue color from the combo stain is achieved. (G) Representative image of PAS‐stained tissue analyzed with AB Intensity (Int) algorithm, no positive intensity (navy blue) represents no PAS signal is being identified with the AB algorithm. (H) Representative image of AB‐stained tissue analyzed with AB Int algorithm, expectation of adequate intensity spectrum is achieved. (I) Representative image of PAS‐AB stained tissue analyzed with AB Int algorithm, expectation of adequate intensity spectrum from the combo stain is achieved. (J) Representative image of PAS‐stained tissue analyzed with PAS CD algorithm, expectation of adequately deconvoluted blue color is achieved. (K) Representative image of AB‐stained tissue analyzed with PAS CD algorithm, no color represents no AB signal is being identified with the PAS algorithm. (L) Representative image of PAS‐AB stained tissue analyzed with PAS CD algorithm, expectation of adequate intensity spectrum from the combo stain is achieved. (M) Representative image of PAS‐stained tissue analyzed with PAS Int algorithm, expectation of adequate intensity spectrum is achieved. (N) Representative image of AB‐stained tissue analyzed with PAS Int algorithm, no positive intensity (navy blue) represents no AB signal is being identified with the PAS algorithm. (O) Representative image of PAS‐AB stained tissue analyzed with PAS Int algorithm, expectation of adequate intensity spectrum from the combo stain is achieved. (P) Representative image of brightfield HID only stain. (Q) Representative image of HID‐stained tissue analyzed with HID CD algorithm, adequately deconvoluted color is achieved. (R) Representative image of adequately measured HID intensity spectrum. (S) Representative image of brightfield SNA and hematoxylin stain. (T) Representative image of SNA‐stained tissue analyzed with SNA CD algorithm, adequately deconvoluted color is achieved. (U) Representative image of adequately measured SNA intensity spectrum. (V) Diagram of the swiss roll technique for ileum tissue (made with Biorender). (W) Intensity spectrum color legend.

**Supplementary Figure S2.** Validation of α‐2,6 N‐SA marker.
Significant digestion of α‐2,6 *N*‐SA with neuraminidase enzyme, negligible digestion of α‐2,3 *N*‐SA. 20x images, Scale bar = 25 μm. A) Representative image of brightfield α‐2,6 *N*‐SA in kidney (SNA + control incubation DiH20). B) Representative image of brightfield α‐2,6 *N*‐SA in kidney (SNA+ α‐2,6 specific neuraminidase). C) Representative image of brightfield α‐2,6 *N*‐SA in kidney (SNA + control incubation DiH20). D) Representative image of brightfield α‐2,6 *N*‐SA in kidney (SNA+ α‐2,3 specific neuraminidase). E) Representative image of brightfield α‐2,6 *N*‐SA in AD tissue (SNA + control incubation DiH20), surrounding an Aβ plaque. F) Representative image of brightfield α‐2,6 *N*‐SA in AD tissue (SNA+ α‐2,6 specific neuraminidase). G) Representative image of brightfield α‐2,6 *N*‐SA in AD tissue (SNA + control incubation DiH20). H) Representative image of brightfield α‐2,6 *N*‐SA in AD tissue (SNA+ α‐2,3 specific neuraminidase).

**Supplementary Figure S3.** Validation of color deconvolution and intensity algorithms within the frontal cortex of a single AD case.
Systematic validation of optimized algorithms surrounding Aβ plaques in the frontal cortex. 20x images, scale bar = 25 μm. A) Representative brightfield image of microglia and phospho tau. B) Representative image of microglia CD. C) Representative image of phospho tau CD. D) Representative image of microglia Int. E) Representative image of phospho tau Int. F) Representative brightfield image of α‐2,6 *N*‐SA. G) Representative image of α‐2,6 *N*‐SA CD. H) Representative image of α‐2,6 *N*‐SA Int. I) Representative brightfield image of Aβ plaque. J) Representative image of Aβ plaque CD. K) Representative image of Aβ plaque Int. L) Representative brightfield image of neutral and sulfonated *O*‐SA. M) Representative image of neutral *O*‐SA CD. N) Representative image of AB sulfonated *O*‐SA CD. O) Representative image of neutral *O*‐SA Int. P) Representative image of sulfonated *O*‐SA Int. Q) Representative brightfield image of HID sulfonated *O*‐SA. R) Representative image of sulfonated *O*‐SA CD. S) Representative image of sulfonated *O*‐SA Int.

**Supplementary Figure S4.** Validation of color deconvolution and intensity algorithms within the hippocampus of a single AD case.
Systematic validation of optimized algorithms surrounding Aβ plaques in the hippocampus. 20x images, scale bar = 25 μm. A) Representative brightfield image of microglia and phospho tau. B) Representative image of microglia CD. C) Representative image of phospho tau CD. D) Representative image of microglia Int. E) Representative image of phospho tau Int. F) Representative brightfield image of α‐2,6 *N*‐SA. G) Representative image of α‐2,6 *N*‐SA CD. H) Representative image of α‐2,6 *N*‐SA Int. I) Representative brightfield image of Aβ plaque. J) Representative image of Aβ plaque CD. K) Representative image of Aβ plaque Int. L) Representative brightfield image of neutral and sulfonated *O*‐SA. M) Representative image of neutral *O*‐SA CD. N) Representative image of AB sulfonated *O*‐SA CD. O) Representative image of neutral *O*‐SA Int. P) Representative image of sulfonated *O*‐SA Int. Q) Representative brightfield image of HID sulfonated *O*‐SA. R) Representative image of sulfonated *O*‐SA CD. S) Representative image of sulfonated *O*‐SA Int.

**Supplementary Figure S5.** Validation of color deconvolution and intensity algorithms within the cerebellum of a single AD case.
Systematic validation of optimized algorithms surrounding Aβ plaques in the cerebellum. 20x images, scale bar = 25 μm. A) Representative brightfield image of microglia. B) Representative image of microglia CD. C) Representative image of microglia Int. D) Representative brightfield image of α‐2,6 *N*‐SA. E) Representative image of α‐2,6 *N*‐SA CD. F) Representative image of α‐2,6 *N*‐SA Int. G) Representative brightfield image of Aβ plaque. H) Representative image of Aβ plaque CD. I) Representative image of Aβ plaque Int. J) Representative brightfield image of neutral and sulfonated *O*‐SA. K) Representative image of neutral *O*‐SA CD. L) Representative image of AB sulfonated *O*‐SA CD. M) Representative image of neutral *O*‐SA Int. N) Representative image of sulfonated *O*‐SA Int. O) Representative brightfield image of HID sulfonated *O*‐SA. P) Representative image of sulfonated *O*‐SA CD. Q) Representative image of sulfonated *O*‐SA Int.

**Supplementary Figure S6.** Holistic representation of all plaque and no plaque ROI values for comparison.
All individual data points represented for plaque and no plaque sialylation quantification. The heterogeneity of data is visible between cases and across regions. The observation of increased *N*‐ and *O*‐SA is visible in the frontal and hippocampus regions analyzed. All graphs represent a Nested 1‐Way ANOVA of percent strong positive signal for plaque and no plaque regions in the 8 cases with amyloid pathology. Multiple comparisons were made across the cases and a chi‐square analysis was completed for general within case sub‐column differences. A) No difference between cases for *N*‐SA in the frontal cortex, but significant sub‐column differences (p < 0.0001). B) No difference between cases or within cases for neutral *O*‐SA in the frontal cortex. C) No difference between cases or within cases for sulfonated *O*‐SA in the frontal cortex. D) No difference between cases for *N*‐SA in the hippocampus, but significant sub‐column differences (p < 0.0001). E) No difference between cases for neutral *O*‐SA in the hippocampus, but significant sub‐column differences (p < 0.0001). F) No difference between cases for neutral *O*‐SA in the hippocampus, but significant sub‐column differences (p = 0.0015).

## Data Availability

Deidentified data sets are included in supplemental data and all other data available upon request.
