# Supplementary material for: Distinct patterns of plaque and microglia glycosylation in Alzheimer's disease
Source: Brain Pathol. 2024 May 9;34(4):e13267. doi: 10.1111/bpa.13267 (PMC11189777; doi:10.1111/bpa.13267)
Supplement: Supplementary file 11 — Data S11. Supporting Information. [file BPA-34-e13267-s016.docx]

Supplemental Methods

**Analysis Validation**

*Positive Control Tissue Validation and Neuraminidase Digestion*

To quantitatively measure positive histological stains for sialylation markers, it is vital to use proper positive controls. Previous work in the field of SA modification use mucosal tissues as standard tissue enriched in glycosylation PTMs. We used murine ileum as our positive control tissue. The ileum was structured in a swiss role configuration to ensure visualization of the intestinal villi structures. We optimized the SNA *N*-SA marker and all 3 *O*-SA markers in this tissue first, evaluating the positive staining in the goblet cells within the ileum (Supplemental Figure 1A-C;P;S). With validated staining in the ileum, the specificity of SNA *N*-SA marker was validated in murine kidney, another tissue known to be enriched in *N*-SA. Using a neuraminidase enzyme specific to the α-2,6 *N*-SA bond (Neuraminidase from Vibrio Cholera Type III; N7885-1UN), tissue was incubated with this enzyme for digestion for 18 hours at 37 degrees Celsius. Technically, a 17.6uL volume of full-strength enzyme was pipetted onto the tissue and a piece of parafilm was placed on top to spread the volume over the tissue and reduce evaporation of the enzyme. After enzyme digestion and control incubation with DiH20, the kidney was stained with the α-2,6 *N*-SA marker (Supplemental Figure 2A;B). The same experiment was performed with a neuraminidase enzyme specific to α-2,3 *N*-SA (Neuraminidase Clostridium Perferingens in 0.1M acetate buffer used at 80mUnit concentration, 52.6uL on tissue; N5631) and kidney tissue was stained with a α-2,6 *N*-SA marker (Supplemental Figure 2C;D). There was a significant reduction in α-2,6 *N*-SA staining in the α-2,6 specific neuraminidase treated tissue compared to the control treated. In contrast, there was not a drastic reduction in α-2,6 *N*-SA after α-2,3 specific neuraminidase digestion. This demonstrates the specificity of our α-2,6 *N*-SA staining. Following validation in kidney, the same protocol was repeated in an AD case and the drastic reduction of α-2,6 *N*-SA is clear surrounding a plaque, the same observation was not made for the α-2,3 specific digestion (Supplemental Figure 2E-H).

*Imagescope Algorithm Validation*

Following validation of histology, ileum control samples were imaged on the Aperio Scanning Microscope and imported into the Imagescope software. Using the color deconvolution v9 default algorithm, each positive control tissue was used to optimize adequate color deconvolution of the known positive control channel (Supplemental figure 1). Specifically, 5 separate algorithms were created to analyze the 5 serial slides. Slide 1 had a dual stain of IBA1 and AT8 and one algorithm was used to analyze the data, with positive channel=3 for IBA1 and positive channel=2 for AT8. Slide 2 had the single stain of α-2,6 SNA on positive channel=3. Slide 3 had the single stain of D54D2 for amyloid plaques with the positive channel=3. Slide 4 was dual stained with PAS-AB, with positive channel=2 for PAS and positive channel=1 for AB. Slide 5 was stained for HID with positive channel=3. Thus, 7 histology markers (IBA1, AT8, SNA, D54D2, PAS, AB, and HID) were evaluated across the 5 slides. See Supplemental Table 1 for all of the thresholds used.

To ensure adequate color deconvolution of the dual stain PAS-AB, validation of color deconvolution was accomplished by running the AB algorithm on the known PAS positive control and ensuring there is no significant color detected (Supp Figure 1D;G). The same process was completed by running the PAS algorithm on the AB positive control (Supp fig 1K;N). This allowed separate color deconvolution of the PAS (magenta) and AB (blue) when stained together. Following validation of color deconvolution, the presence positive signal was optimized on the positive control tissues. To ensure proper visualization of the stain, color deconvolution must be correct. To achieve the proper positive signal measurements, we utilized the tune feature to toggle between brightfield and thresholding readout to ensure the strongest positive staining is measuring as strong positive (red), medium positive (orange), weak positive (yellow), and non-positive signal (navy). See supplemental figure 1 for examples of all thresholded readouts for each positive control ileum. After validation of the algorithms in the ileum, the algorithms were checked on plaque regions across the 3 regions sampled (Supplemental Figures 3,4,5). Then, each optimized algorithm was saved to be used on the experimental slides.

**Image Analysis**

*Cohort Analysis of Aβ Plaques and No Plaque Regions*

In this analysis, 5 paired Aβ plaque regions and adjacent no plaque regions were sampled for all cases in the 3 brain regions. These ROIs were paired. Additionally, the 5 plaque and 5 no plaque ROIs was synced across all 5 serial slides in each region of each case. This technique of synced ROIs ensures the ability to compare histologic stains across all slides in the same region. For this analysis, the uniform ROI was 100x100x100µm in size. The anatomical regions analyzed was the cortical ribbon of the middle frontal gyrus, CA1 and subiculum of the hippocampus, and the molecular layer of the cerebellar dentate. The output measures used for analysis included the percentage of strong positive histologic staining, defined as the percent strong positive signal. Comparisons of sialylation differences between plaque and no plaque regions were accomplished with 2-Way Mixed Effects ANOVAs. This form of ANOVA was used because the data was not distributed normally. N-sialylation was compared with 2-Way ANOVA of average percent strong positive α-2,6 SNA signal (plaque pathology- plaque vs no plaque- x region- frontal, hippocampus, and cerebellum). Pairwise comparisons were made for within region differences (ie. frontal plaque vs no plaque) and between region difference (ie. frontal plaque vs hippocampus plaque). The same analyses were completed for PAS average strong positive signal and AB average strong positive signal. All data is presented as mean with standard deviation (SD).

*Quantification of Sialylated Microglia*

To investigate the distribution of *N*-sialylated microglia within the plaque and no plaque regions, cell counts were conducted within each 100x100x100µm ROI in the frontal cortex. Using the IBA1 optimized deconvolution algorithm, the IBA1 positive signal was used to manually count the number of IBA1 positive microglia. Then, in the adjacent SNA stain, the SNA algorithm was used to identify SNA positive microglia. Comparing the two slides, the number of SNA positive microglia that were also IBA1 positive were counted. Following manual counting, the percentage of sialylated microglia was calculated buy [(count of SNA positive microglia that are also IBA1 positive/count of IBA1 positive microglia) x100]. In the 8 cases with Aβ pathology in the frontal cortex, a paired T-test was to compare all percent sialylated microglia across the plaque and no plaque regions. To investigate the difference in sialylated microglia in low pathology cases compared to high pathology cases, a non-parametric T-test was used. To understand the percent of sialylated microglia and plaque pathology, the percent strong positive IBA1 and percent strong positive SNA staining were compared in a 2-Way Mixed Effects ANOVA (plaque pathology x histology stain). All data is presented as mean with SD.

*Quantification of Sialylation Relative to Aβ Plaques Morphology*

To understand the sialylation landscape relative to Aβ plaques, the comparison of plaque morphologies was necessary. For this analysis, three paired 100x100x100µm plaque and no plaque ROIs were used across the frontal cortex sections of the 8 cases with amyloid pathology. Within the plaque ROI category, 3 ROIs for cored and 3 for diffuse plaque morphologies were identified. The average percent strong positive signal of each histological stain in the plaque and no plaque regions was quantified with a 2-Way Mixed Effects ANOVA (histologic stain x plaque morphology). To compare the *N*- and *O*-linked sialylation contributions to Aβ plaque morphologies, percent strong positive signal (percent area) of each SA stain were used for localization. The average percent area of SNA, PAS, AB, and HID were compared in a 2-Way Mixed Effects ANOVA (histologic stain x plaque morphology). All data is presented as mean with SD.

*Quantification of Sialylation Relative to Tau Pathology*

In this analysis, the comparison of high tau pathology and low pathology burden were investigated. The CA2 region of the hippocampus was defined as a high tau pathology region with little Aβ plaque contribution in these cases. Within the CA2, the ROIs captured neurofibrillary tangle pathology. The CA4 region of the hippocampus was defined as a lower tau pathology region within these cases. This analysis utilized one paired 200x200x200µm ROI across all 5 serial slides for the 8 cases with tau pathology in the hippocampus. To compare the association of tau pathology and sialylation markers, a 2-Way Repeated Measures ANOVA (histologic stain x tau pathology burden) was used with SD for variance. IF images were imported into Image J image processing software with Fiji version 1.53q. To quantitatively determine the location of α-2,6 SA relative to tau, a pixel-to-pixel correlation was performed for correlation pairs of SA-to-tau. Pearson’s correlation was employed to analyze pixel-to-pixel ratios using above value thresholds only (SNA: Li 8638/26450 & AT8: Moments 3740/default). To present this data, Pearson correlation coefficient r values were used to represent the strength and direction of the relationship between the two variables (SA to tau). The r values were then compared with a paired T-test.

Supplementary Table 1

| **Target** | **Color Deconvolution/Thresholds** |
| --- | --- |
| Microglia (IBA1) | Positive Channel 3: Red=0.31, Green=0.552, Blue=0.776; Int=99, 173, 218 |
| Tau (AT8) | Positive Channel 2: Red=0.288, Green=0.99, Blue=0.671; Int=99, 173, 218 |
| N-Linked SA (SNA) | Positive Channel 3: Red=0.31, Green=0.552, Blue=0.776; Int=93,163,208 |
| Aβ Plaques (D54D2) | Positive Channel 3: Red=0.31, Green=0.552, Blue=0.776; Int=77, 175, 220 |
| Neutral O-linked SA (PAS) | Positive Channel 2: Red=0.265, Green=0.704, Blue=0.228; Int=168,183,220 |
| Sulfonated O-linked SA (AB) | Positive Channel 1: Red=0.33, Green=0.175, Blue=0.265; Int=168,183,220 |
| Sulfonated O-linked SA (HID) | Positive Channel 3: Red=0.22, Green=0.257, Blue=0.635; Int=153, 190, 217 |
