# Supplementary figures and images for: Distinct patterns of plaque and microglia glycosylation in Alzheimer's disease
Source: Brain Pathol. 2024 May 9;34(4):e13267. doi: 10.1111/bpa.13267 (PMC11189777; doi:10.1111/bpa.13267)

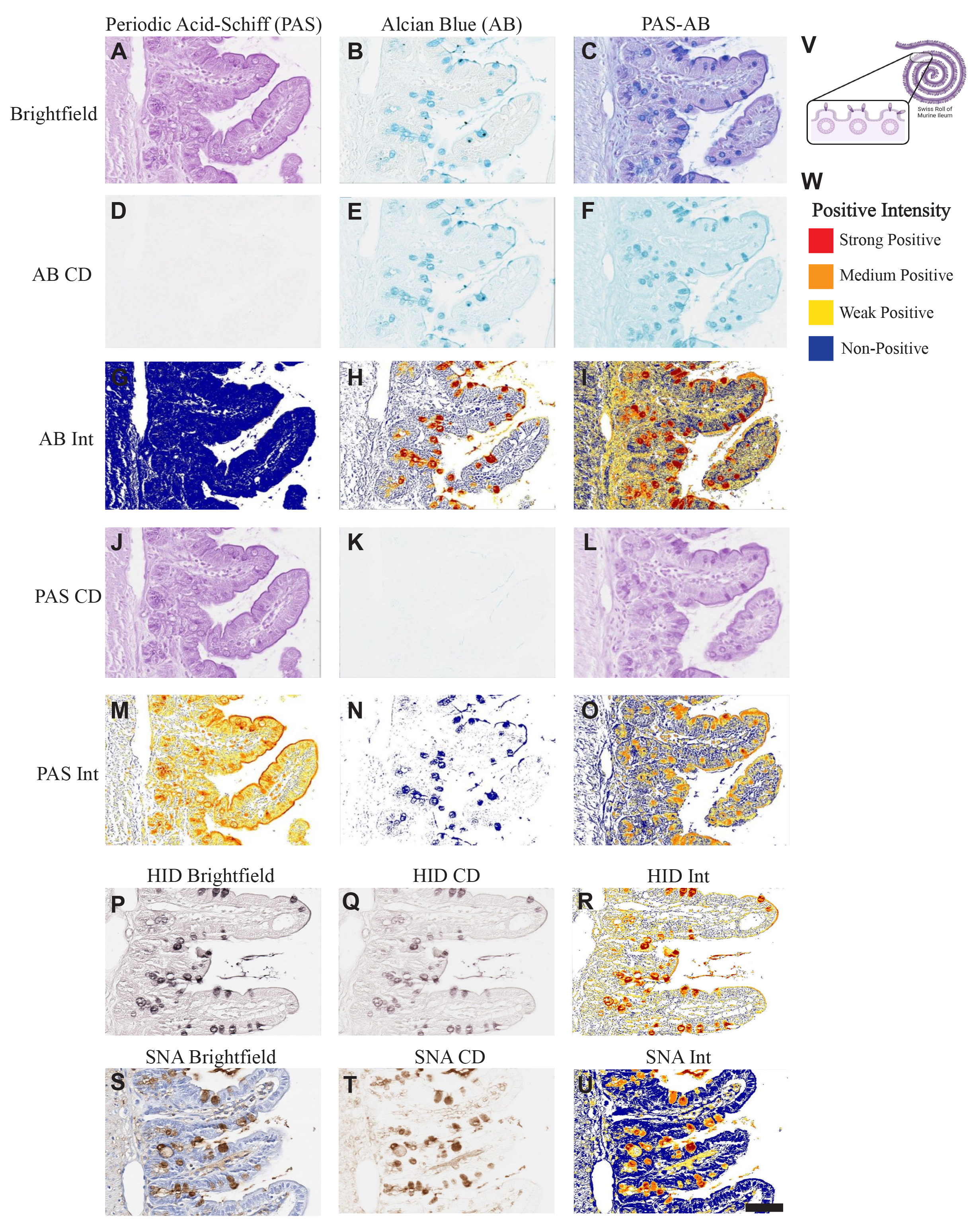

Supplement: Supplementary file 12 — Supplementary Figure S1. Imagescope algorithm validation on positive control tissue. Systematic validation of O‐ and N‐SA makers utilizing Imagescope color deconvolution (CD) algorithm. 20x images, Scale bar = 25 μm. (A) Representative image of brightfield PAS only stain. (B) Representative image of brightfield AB only stain. (C) Representative image of brightfield PAS‐AB combo stain. (D) Representative image of PAS‐stained tissue analyzed with AB CD algorithm, no color represents no PAS signal is being identified with the AB algorithm. (E) Representative image of AB‐stained tissue analyzed with AB CD algorithm, expectation of adequately deconvoluted blue color is achieved. (F) Representative image of PAS‐AB stained tissue analyzed with AB CD algorithm, expectation of adequately deconvoluted blue color from the combo stain is achieved. (G) Representative image of PAS‐stained tissue analyzed with AB Intensity (Int) algorithm, no positive intensity (navy blue) represents no PAS signal is being identified with the AB algorithm. (H) Representative image of AB‐stained tissue analyzed with AB Int algorithm, expectation of adequate intensity spectrum is achieved. (I) Representative image of PAS‐AB stained tissue analyzed with AB Int algorithm, expectation of adequate intensity spectrum from the combo stain is achieved. (J) Representative image of PAS‐stained tissue analyzed with PAS CD algorithm, expectation of adequately deconvoluted blue color is achieved. (K) Representative image of AB‐stained tissue analyzed with PAS CD algorithm, no color represents no AB signal is being identified with the PAS algorithm. (L) Representative image of PAS‐AB stained tissue analyzed with PAS CD algorithm, expectation of adequate intensity spectrum from the combo stain is achieved. (M) Representative image of PAS‐stained tissue analyzed with PAS Int algorithm, expectation of adequate intensity spectrum is achieved. (N) Representative image of AB‐stained tissue analyzed with PAS Int algori [file BPA-34-e13267-s014.tif]

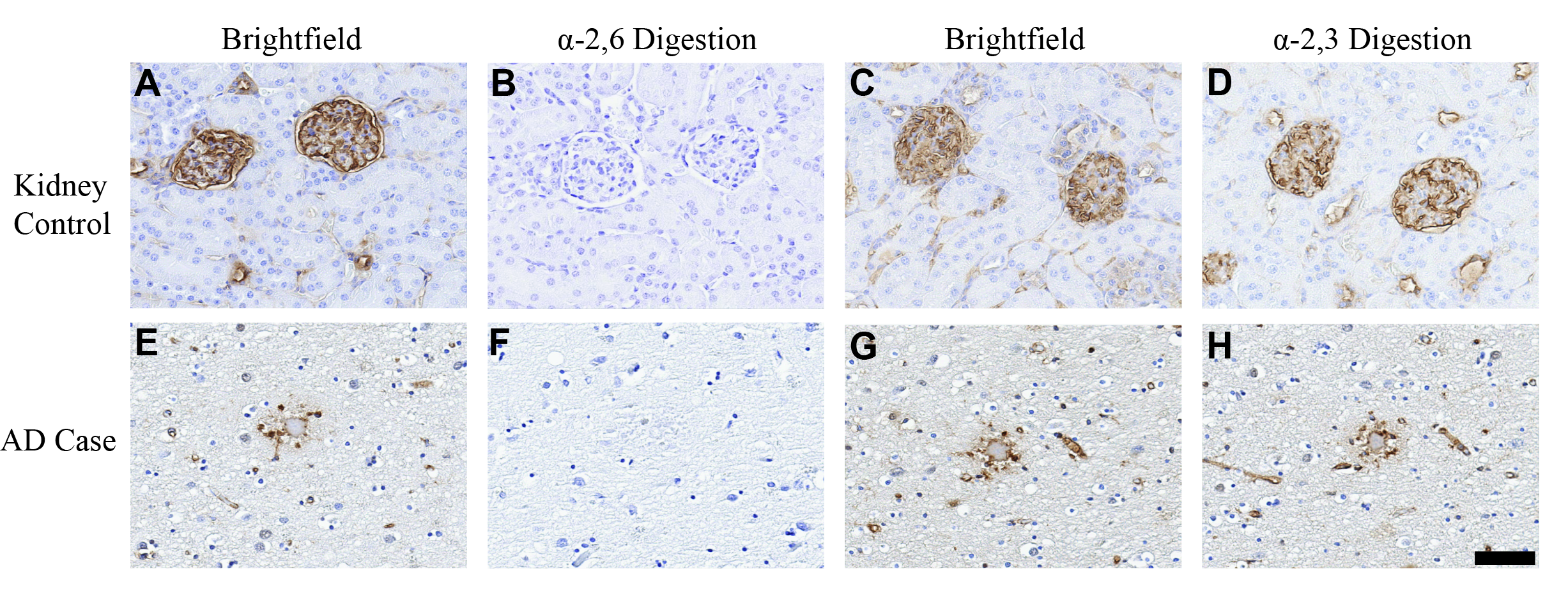

Supplement: Supplementary file 13 — Supplementary Figure S2. Validation of α‐2,6 N‐SA marker. Significant digestion of α‐2,6 N‐SA with neuraminidase enzyme, negligible digestion of α‐2,3 N‐SA. 20x images, Scale bar = 25 μm. A) Representative image of brightfield α‐2,6 N‐SA in kidney (SNA + control incubation DiH20). B) Representative image of brightfield α‐2,6 N‐SA in kidney (SNA+ α‐2,6 specific neuraminidase). C) Representative image of brightfield α‐2,6 N‐SA in kidney (SNA + control incubation DiH20). D) Representative image of brightfield α‐2,6 N‐SA in kidney (SNA+ α‐2,3 specific neuraminidase). E) Representative image of brightfield α‐2,6 N‐SA in AD tissue (SNA + control incubation DiH20), surrounding an Aβ plaque. F) Representative image of brightfield α‐2,6 N‐SA in AD tissue (SNA+ α‐2,6 specific neuraminidase). G) Representative image of brightfield α‐2,6 N‐SA in AD tissue (SNA + control incubation DiH20). H) Representative image of brightfield α‐2,6 N‐SA in AD tissue (SNA+ α‐2,3 specific neuraminidase). [file BPA-34-e13267-s010.tif]

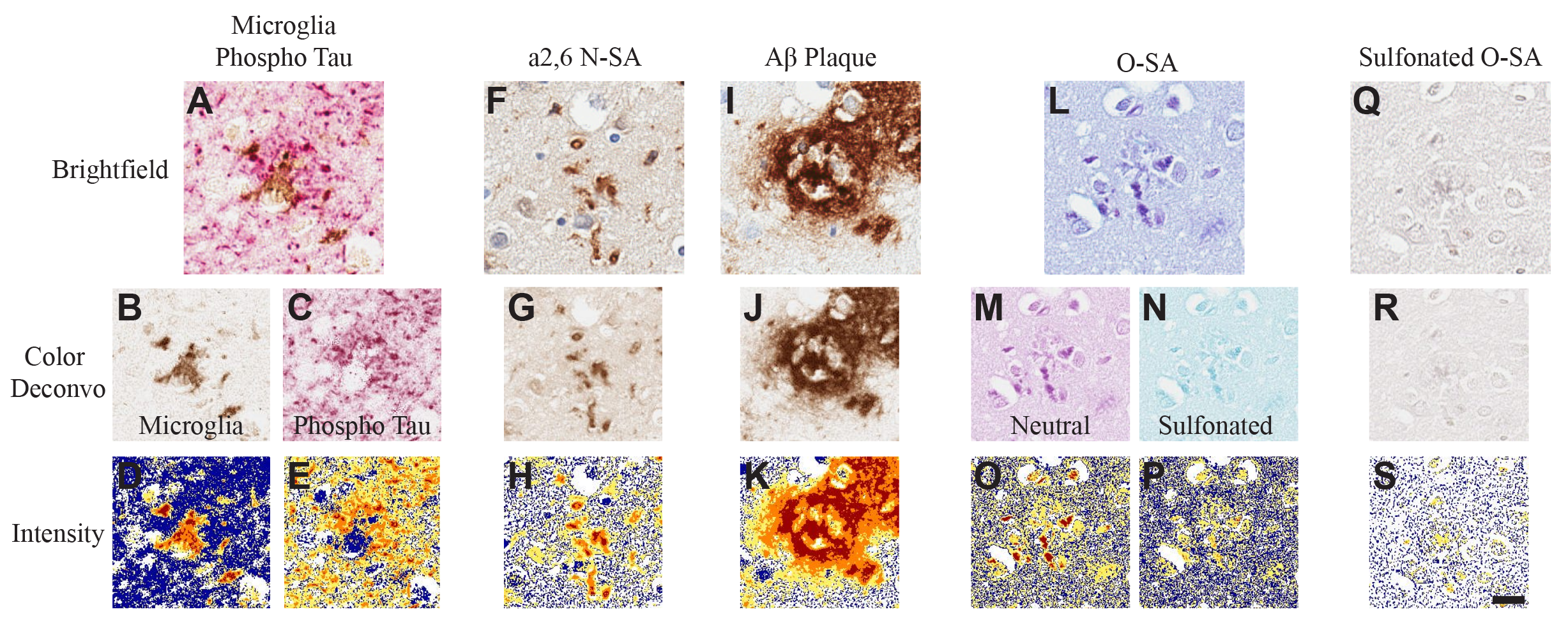

Supplement: Supplementary file 14 — Supplementary Figure S3. Validation of color deconvolution and intensity algorithms within the frontal cortex of a single AD case. Systematic validation of optimized algorithms surrounding Aβ plaques in the frontal cortex. 20x images, scale bar = 25 μm. A) Representative brightfield image of microglia and phospho tau. B) Representative image of microglia CD. C) Representative image of phospho tau CD. D) Representative image of microglia Int. E) Representative image of phospho tau Int. F) Representative brightfield image of α‐2,6 N‐SA. G) Representative image of α‐2,6 N‐SA CD. H) Representative image of α‐2,6 N‐SA Int. I) Representative brightfield image of Aβ plaque. J) Representative image of Aβ plaque CD. K) Representative image of Aβ plaque Int. L) Representative brightfield image of neutral and sulfonated O‐SA. M) Representative image of neutral O‐SA CD. N) Representative image of AB sulfonated O‐SA CD. O) Representative image of neutral O‐SA Int. P) Representative image of sulfonated O‐SA Int. Q) Representative brightfield image of HID sulfonated O‐SA. R) Representative image of sulfonated O‐SA CD. S) Representative image of sulfonated O‐SA Int. [file BPA-34-e13267-s005.tif]

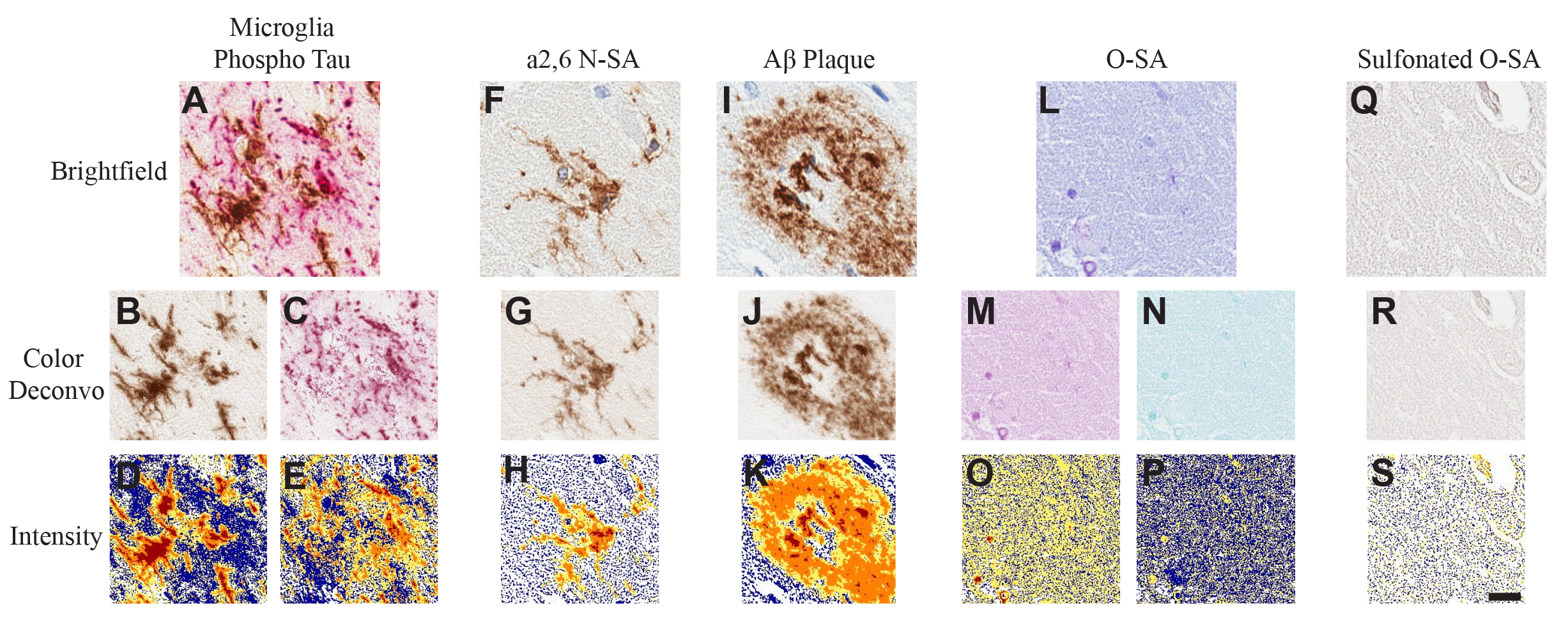

Supplement: Supplementary file 15 — Supplementary Figure S4. Validation of color deconvolution and intensity algorithms within the hippocampus of a single AD case. Systematic validation of optimized algorithms surrounding Aβ plaques in the hippocampus. 20x images, scale bar = 25 μm. A) Representative brightfield image of microglia and phospho tau. B) Representative image of microglia CD. C) Representative image of phospho tau CD. D) Representative image of microglia Int. E) Representative image of phospho tau Int. F) Representative brightfield image of α‐2,6 N‐SA. G) Representative image of α‐2,6 N‐SA CD. H) Representative image of α‐2,6 N‐SA Int. I) Representative brightfield image of Aβ plaque. J) Representative image of Aβ plaque CD. K) Representative image of Aβ plaque Int. L) Representative brightfield image of neutral and sulfonated O‐SA. M) Representative image of neutral O‐SA CD. N) Representative image of AB sulfonated O‐SA CD. O) Representative image of neutral O‐SA Int. P) Representative image of sulfonated O‐SA Int. Q) Representative brightfield image of HID sulfonated O‐SA. R) Representative image of sulfonated O‐SA CD. S) Representative image of sulfonated O‐SA Int. [file BPA-34-e13267-s003.tif]

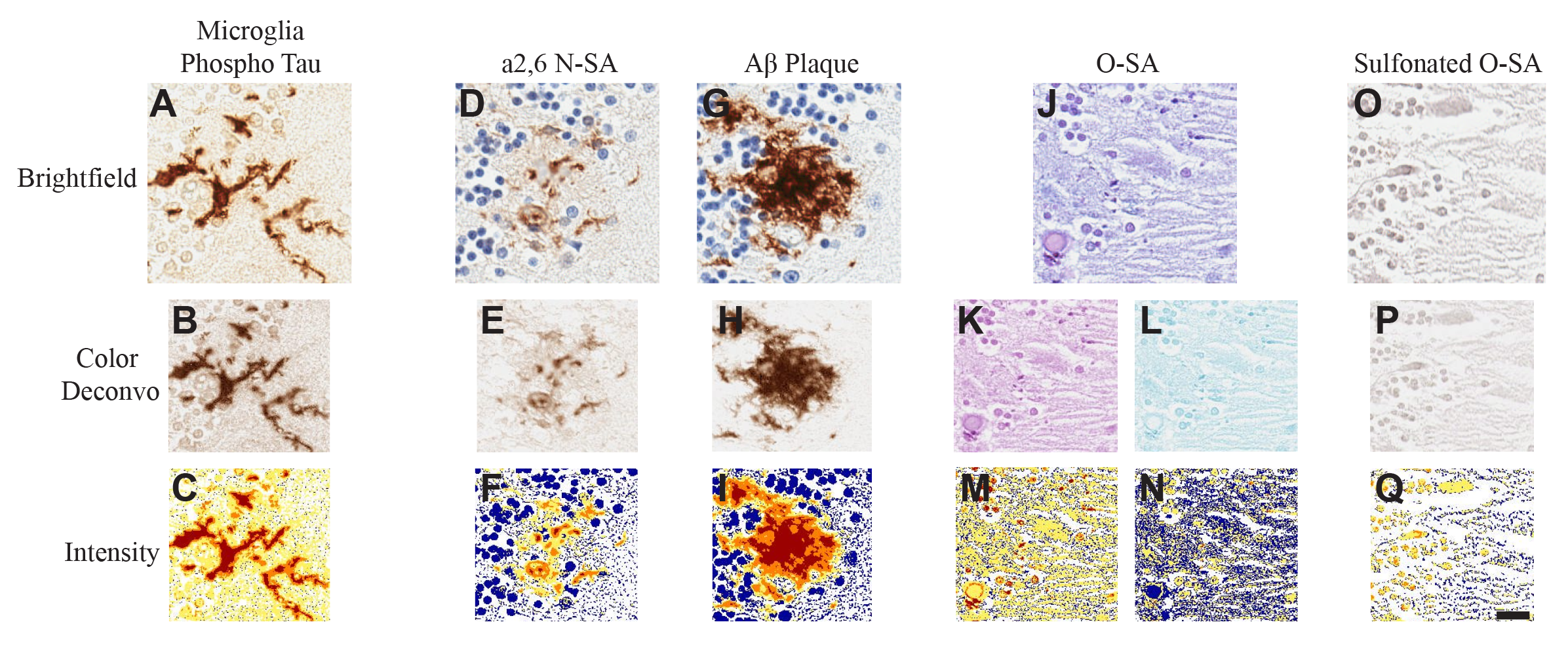

Supplement: Supplementary file 16 — Supplementary Figure S5. Validation of color deconvolution and intensity algorithms within the cerebellum of a single AD case. Systematic validation of optimized algorithms surrounding Aβ plaques in the cerebellum. 20x images, scale bar = 25 μm. A) Representative brightfield image of microglia. B) Representative image of microglia CD. C) Representative image of microglia Int. D) Representative brightfield image of α‐2,6 N‐SA. E) Representative image of α‐2,6 N‐SA CD. F) Representative image of α‐2,6 N‐SA Int. G) Representative brightfield image of Aβ plaque. H) Representative image of Aβ plaque CD. I) Representative image of Aβ plaque Int. J) Representative brightfield image of neutral and sulfonated O‐SA. K) Representative image of neutral O‐SA CD. L) Representative image of AB sulfonated O‐SA CD. M) Representative image of neutral O‐SA Int. N) Representative image of sulfonated O‐SA Int. O) Representative brightfield image of HID sulfonated O‐SA. P) Representative image of sulfonated O‐SA CD. Q) Representative image of sulfonated O‐SA Int. [file BPA-34-e13267-s008.tif]

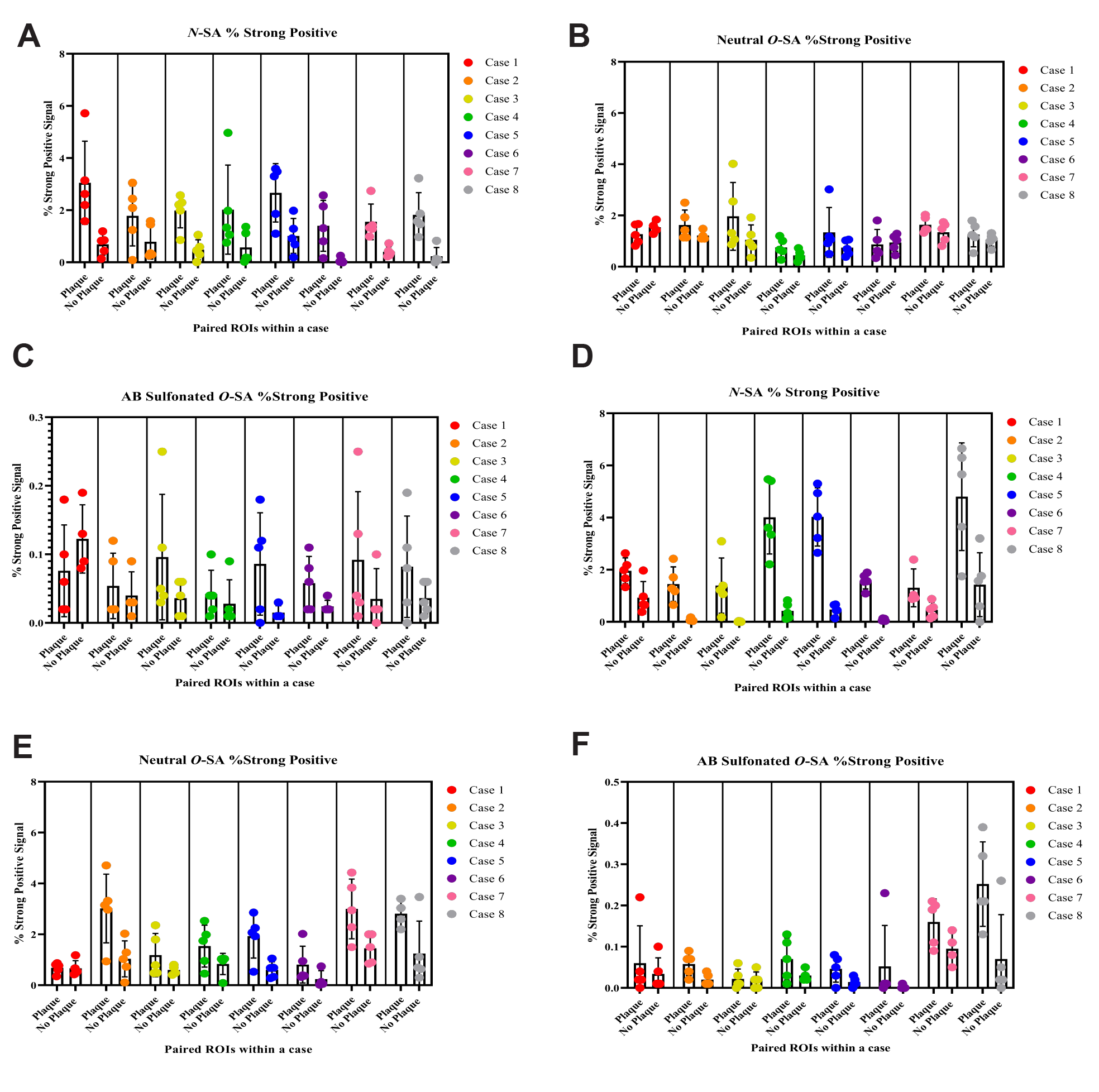

Supplement: Supplementary file 17 — Supplementary Figure S6. Holistic representation of all plaque and no plaque ROI values for comparison. All individual data points represented for plaque and no plaque sialylation quantification. The heterogeneity of data is visible between cases and across regions. The observation of increased N‐ and O‐SA is visible in the frontal and hippocampus regions analyzed. All graphs represent a Nested 1‐Way ANOVA of percent strong positive signal for plaque and no plaque regions in the 8 cases with amyloid pathology. Multiple comparisons were made across the cases and a chi‐square analysis was completed for general within case sub‐column differences. A) No difference between cases for N‐SA in the frontal cortex, but significant sub‐column differences (p < 0.0001). B) No difference between cases or within cases for neutral O‐SA in the frontal cortex. C) No difference between cases or within cases for sulfonated O‐SA in the frontal cortex. D) No difference between cases for N‐SA in the hippocampus, but significant sub‐column differences (p < 0.0001). E) No difference between cases for neutral O‐SA in the hippocampus, but significant sub‐column differences (p < 0.0001). F) No difference between cases for neutral O‐SA in the hippocampus, but significant sub‐column differences (p = 0.0015). [file BPA-34-e13267-s013.tif]
